# Supplementary material for: Machine Learning Made Easy (MLme): a comprehensive toolkit for machine learning–driven data analysis
Source: Gigascience. 2024 Jan 11;13:giad111. doi: 10.1093/gigascience/giad111 (PMC10783149; doi:10.1093/gigascience/giad111)
Supplement: giad111_GIGA-D-23-00182_Revision_1 [file giad111_giga-d-23-00182_revision_1.pdf]

GigaScience

Machine Learning Made Easy (MLme): A Comprehensive Toolkit for Machine Learning-Driven Data Analysis

--Manuscript Draft--

**Manuscript Number:**	GIGA-D-23-00182R1
**Full Title:**	Machine Learning Made Easy (MLme): A Comprehensive Toolkit for Machine Learning-Driven Data Analysis
**Article Type:**	Technical Note

Funding Information:

Schweizerischer Nationalfonds zur Förderung der Wissenschaftlichen Forschung (310030_175773)	Prof Fiona C Burkhard
Schweizerischer Nationalfonds zur Förderung der Wissenschaftlichen Forschung (212298)	Dr Ali Hashemi Gheinani
Else Kröner-Fresenius-Zentrum für Ernährungsmedizin (EKFS 2021_EKeA.33)	Ms Mitali Katoch
Sächsisches Staatsministerium für Wissenschaft und Kunst (ScaDS.AI)	Mr Masoud Abedi

Abstract:

Background

Machine learning (ML) has emerged as a vital asset for researchers to analyze and extract valuable information from complex datasets. However, developing an effective and robust ML pipeline can present a real challenge, demanding considerable time and effort, thereby impeding research progress. Existing tools in this landscape require a profound understanding of ML principles and programming skills. Furthermore, users are required to engage in the comprehensive configuration of their ML pipeline to obtain optimal performance.

Results

To address these challenges, we have developed a novel tool called Machine Learning Made Easy (MLme) that streamlines the use of ML in research, specifically focusing on classification problems at present. By integrating four essential functionalities, namely Data Exploration, AutoML, CustomML, and Visualization, MLme fulfills the diverse requirements of researchers while eliminating the need for extensive coding efforts. To demonstrate the applicability of MLme, we conducted rigorous testing on six distinct datasets, each presenting unique characteristics and challenges. Our results consistently showed promising performance across different datasets, reaffirming the versatility and effectiveness of the tool. Additionally, by utilizing MLme's feature selection functionality, we successfully identified significant markers for CD8+ naive (BACH2), CD16+ (CD16), and CD14+ (VCAN) cell populations.

Conclusion

MLme serves as a valuable resource for leveraging machine learning (ML) to facilitate insightful data analysis and enhance research outcomes, while alleviating concerns related to complex coding scripts. The source code and a detailed tutorial for MLme are available at https://github.com/FunctionalUrology/MLme.

**Corresponding Author:**	Ali Hashemi Gheinani<br>Boston Children's Hospital<br>SWITZERLAND
**Corresponding Author Secondary Information:**	
**Corresponding Author's Institution:**	Boston Children's Hospital

Powered by Editorial Manager® and ProduXion Manager® from Aries Systems Corporation

**Corresponding Author's Secondary Institution:**	
**First Author:**	Akshay Akshay
**First Author Secondary Information:**	
**Order of Authors:**	Akshay Akshay
	Mitali Katoch
	Navid Shekarchizadeh
	Masoud Abedi
	ankush Sharma
	Fiona C Burkhard
	Rosalyn M Adam
	Katia Monastyrskaya
	Ali Hashemi Gheinani
**Order of Authors Secondary Information:**	
**Response to Reviewers:**	Dear Editors,<br><br>I would like to express our sincere gratitude for the valuable comments provided by the reviewers of our manuscript submitted to Gigascience Journal. Their constructive feedback has been instrumental in enhancing the quality of our work, and we are grateful for their time and insights.<br><br>We have carefully addressed the reviewers' comments and made significant revisions to the manuscript to incorporate their suggestions. Specifically, we have registered our software application in the bio.tools and SciCrunch.org databases, as requested, and have included the corresponding RRID and biotoolsID identifiers in the manuscript, as outlined in the reviewers' feedback.<br><br>We have also responded comprehensively to each of the reviewers' major and minor concerns, providing detailed explanations and making the necessary adjustments to our manuscript and software where applicable. We have included additional comparisons with other tools, expanded the feature descriptions, and improved transparency regarding our pipeline's functionalities.<br><br>Furthermore, we have taken the reviewer's suggestions to heart and have removed the default data resampling option, providing users with the ability to choose whether to include it in their workflow. We believe this adjustment enhances transparency and user control.<br><br>Additionally, we have included a larger dataset to showcase MLme's versatility and applicability across various data sizes and types.<br><br>Once again, we would like to express our appreciation for the reviewers' efforts in evaluating our work. We believe that the revisions we have made address their concerns and strengthen our manuscript. We are confident that these improvements will enhance the value of our contribution to the field of machine learning-driven analysis.<br><br>Thank you for your consideration.<br>Ali Hashemi<br><br>-------------------------------------- Editor's Comment<br>In addition, please register any new software application in the bio.tools and SciCrunch.org databases to receive RRID (Research Resource Identification Initiative ID) and biotoolsID identifiers, and include these in your manuscript. Computational workflows should be registered in workflowhub.eu and the DOIs cited in the relevant places in the manuscript. These will facilitate tracking, reproducibility and re-use of

Powered by Editorial Manager® and ProduXion Manager® from Aries Systems Corporation

your tool.

.........................Response to Editor's Comment ....
Thank you for your valuable feedback. In compliance with your request, we have included these unique identifiers in the manuscript. They can be found at line 401 and 402 in main manuscript under Availability of supporting source code and requirements.

BioTool ID: MLme
https://bio.tools/mlme

SciCrunch ID: MLme (RRID: SCR_024439)
https://scicrunch.org/resources/about/registry/SCR_024439

Workflow: https://workflowhub.eu/workflows/571?version=1
(cited in line number 189)

##
Reviewer 1
##

--------------------------------------- Reviewer's Comment
Reviewer #1: Akshay et al. present MLme, a toolkit for exploring data and automatically running machine learning (ML) models. This software could be useful for those with less experience in ML. I believe it is suitable for publication provided the following points are addressed.

.........................Response to Reviewer's Comment ....
Thank you for your time and thoughtful feedback on our manuscript about MLme. Your contribution has been instrumental in improving the quality of our manuscript, and we greatly appreciate your insights. Below, you will find our responses to each of your comments.

Major

--------------------------------------- Reviewer's Comment

1. The performance of models is consistently over 90% but without a reference point it is unclear how good this is. Are there results from previous studies on the same data that can be compared to, with a table comparing accuracy with MLme to previous work? Otherwise it is unclear whether MLme is supposed to be a quick way to have a first go at prediction on the data or can entirely replace manual model refinement.

.........................Response to Reviewer's Comment ....
Thank you for your valuable input. We appreciate your suggestion and have taken it into careful consideration. We have conducted a comparison between MLme, TPOT, and hyperopt-sklearn, and as expected, the results from both tools demonstrate comparability. For specific details and results, please refer to lines 307 to 315 and Figure S9.C . We believe this additional information will provide valuable insights to our readers.

---------------------------------------Reviewer's Comment
2. With any automated ML system it is important to impress upon users the risks of ML. For example, the splitting of data into training and test sets is done randomly, but there are cases where this is not appropriate as it will lead to data leakage between the training and test sets. This could be mentioned in the manuscript and somewhere on the GUI. There isn't really a replacement for domain knowledge, and users of MLme should have this in mind when using the software.
.........................Response to Reviewer's Comment ....
Thank you for your feedback and valuable suggestion regarding the inclusion of

Powered by Editorial Manager® and ProduXion Manager® from Aries Systems Corporation

information on the risks associated with the automated ML tools. We have taken your input seriously, and we are pleased to inform you that we have incorporated the corresponding text into the Conclusion section of our manuscript, spanning from line 371 to 376.

Minor
---------------------------------------Reviewer's Comment
3. More experienced ML users may want to use the software to have a first go at prediction on the data. For these users it may be useful to provide access to commands or scripts, or at least information on which functions were used, as additional options in the GUI. Users could then run these scripts themselves to tweak hyperparameters etc.

.........................Response to Reviewer's Comment ....
Certainly, we appreciate your interest in enhancing the utility of MLme! We are pleased to inform you that we have already made the autoML script publicly available on the MLme GitHub repository (https://github.com/FunctionalUrology/MLme/blob/main/subScript.py). Additionally, CustomML also provides the scripts, a readme file, and allows users to select values for each hyperparameter of all the available algorithms.

---------------------------------------Reviewer's Comment
4. The visualisation tab lacks an info button by the file upload to say what the file format should be.
.........................Response to Reviewer's Comment ....
Thank you for bringing this to our attention. We have made an update to the visualization tab. An "info" button has been added next to the file upload section to provide clear guidance on the acceptable file formats.

##
Reviewer 2
##

---------------------------------------Reviewer's Comment
Reviewer #2: In this paper the authors introduce MLme, a comprehensive toolkit for machine-learning driven analysis. The authors discuss the benefits and limitations of their toolkit and provide a demonstration evaluation on 6 datasets suggesting it's potential value. Overall MLme seems like a nice, easy to use tool with a good deal of potential and value.

.........................Response to Reviewer's Comment ....
Thank you for your time and thoughtful feedback on our manuscript about MLme. Your contribution has been instrumental in improving the quality of our manuscript, and we greatly appreciate your insights. Below, you will find our responses to each of your comments.

---------------------------------------Reviewer's Comment
- However, as the developer of STREAMLINE, an AutoML toolkit with a number of very similar goals and design architecture to MLme it was very surprising to have it not referenced or compared to in this paper. My major concerns involve the limited details about what this specifically does/includes (e.g. what 16 ML algorithms are built in), as well as what seems like a limited and largely biased comparison of this toolkit's capabilities to other autoML tools (most specifically STREAMLINE which has a significant degree of similarity).

.........................Response to Reviewer's Comment ....
We appreciate the reviewer for bringing STREAMLINE to our attention and for taking

the time to provide valuable and constructive feedback.
Indeed, STREAMLINE and MLme share common goals and address similar problems in the field of ML. We acknowledge that STREAMLINE offers some important features, such as data imputation and automatic hyperparameter tuning, which are currently not available in MLme. We appreciate the opportunity to learn about these features and will consider them for potential future enhancements to our toolkit.
One of the distinctive features of MLme is its emphasis on providing users with a code-free experience while offering extensive control over the design of machine learning pipelines. This feature sets MLme apart from other AutoML tools, including STREAMLINE. Additionally, we would like to emphasize that MLme is not solely an AutoML tool; it is a comprehensive platform encompassing data exploration, AutoML, custom ML pipeline design, and result visualization. All these functionalities are designed to be user-friendly and interactive, facilitated by an intuitive user interface. With these features, MLme aims to cater to users with limited programming experience, providing accessibility to machine learning for those who may not possess advanced coding skills. Even for experienced users, there is a steep learning curve to use the other available tools. While STREAMLINE also strives to achieve this goal to some extent, it still requires users to have coding expertise.
Initially, we did not include tools in our comparison table (S1) that demand programming expertise, with the exceptions of Hyperopt and Autosklearn, due to their popularity in the field. However, in response to your comment, we have endeavored to provide a fair and comprehensive comparison between MLme and the other tools, as detailed in table S1.
For a more detailed understanding of the algorithms and steps integrated into MLme, please refer to figure S10 (line 235-237).

--------------------------------------Reviewer's Comment
- There are many other autoML tools out there that that authors have not considered in their Table S1 or referenced. Eg. MLBox, AutoWeka, H20, Devol, Auto-Keras, TransmorgriffAI, and most glaringly in for this reviewer, STREAMLINE (https://github.com/UrbsLab/STREAMLINE).

.........................Response to Reviewer's Comment ....
We value the feedback from the reviewers and believe that the expanded Table S1 has effectively addressed your concerns.
We would like to mention that, Devol is not an autoML tool but rather a tool for automatically building neural networks. Therefore, it cannot be compared to MLme or other tools mentioned here.

--------------------------------------Reviewer's Comment
- In particular, with respect to STREAMLINE (https://link.springer.com/chapter/10.1007/978-981-19-8460-0_9), there are a large number of pipeline similarities and a similar analysis mission/goals to MLme that make it extremely relevant to cite and contrast to in this manuscript as well as in Table S1. STREAMLINE has a similar focus on the end-to-end ML analysis pipeline including automated exploratory analysis, data processing, feature selection, ML modeling with 16 algorithms, evaluation, and results visualization generation, interactive visualizations, pickled output storage, etc. The first STREAMLINE paper was published March of 2023, and a preprint of that manuscript published June 2022, as well as a precursor implementation of this pipeline published as a preprint in Aug of 2020 (https://arxiv.org/abs/2008.12829). This in contrast with MLme's preprint published July of 2023. While MLme has a number of potentially nice features that STREAMLINE does not (i.e. a GUI interface, spider plots, easy color palate selection, inclusion of a dummy classifier, ability to handle multi-class classification [which is not yet available, but in development for STREAMLINE along with regression]), it lacks other potentially important features that STREAMLINE does have (i.e. automated hyperparameter optimization, basic data cleaning and feature engineering [in the newest release], collective feature selection, pickled models for later reuse, collective feature importance visualizations, a pdf analysis summary report, the ability to quickly evaluate models on new replication data, and potentially other capabilities that I can't highlight because of limited details on what MLme includes). The absence of hyperparameter optimization is a particularly problematic omission from MLme, as this a fairly critical

element of any machine learning analysis pipeline.

.........................Response to Reviewer's Comment ....
We sincerely appreciate your thorough review and valuable insights.
Regarding the absence of automated feature engineering in MLme, we firmly believe in the importance of domain expertise in this process. Different datasets and domains often require tailored feature processing approaches. For instance, in proteomics datasets, where missing values are prevalent, a simplistic mean/median imputation might not suffice. We believe that users should have control over these crucial decisions to ensure the best outcomes. This approach empowers users to make informed choices based on their specific data characteristics and domain knowledge.
Regarding hyperparameter optimization, we acknowledge its significance in machine learning pipelines. This feature is slated for future updates, as outlined in our manuscript (lines 385 to 391). In the current version of MLme, users can choose values for every parameter across all the available algorithms.
Furthermore, MLme offers a range of functionalities, including feature selection, pickled models for future use and evaluation on new data, and interactive result visualizations. Our visualizations are designed to be dynamic and publication-ready, providing users with a more interactive and informative experience compared to static reports.
Once again, we appreciate your feedback, which will undoubtedly help us enhance MLme's capabilities and usability for the research community.

---------------------------------------Reviewer's Comment
-Table S1 should be expanded to highlight a broader range of toolkit features to better highlight the strengths and weaknesses of a greater variety of methodologies. The present table seems a bit cherry picked to make MLme stand out as appearing to have more capabilities than other tools, but there are uncaptured advantages to these other approaches.

.........................Response to Reviewer's Comment ....
Thank you for your feedback. We have attempted to address this by expanding Table S1.

---------------------------------------Reviewer's Comment
-This manuscript includes no citations justifying their pipeline design choices. In particular, I'm most concerned with the author's justification of automatically including data resampling by default as it is well known that this can introduce bias in modeling. It's also not clear what determines if data resampling is required, and whether this only impacts training data or also testing data. Its not clear that resampling is a good/reliable strategy for an automated machine learning framework since data resampling to create a more balanced dataset can also incorporate bias in to an ML model.

.........................Response to Reviewer's Comment ....
Thank you for your thoughtful feedback, and we appreciate your comments regarding the inclusion of data resampling by default.
Initially, our intention was to simplify the user experience in our autoML feature by limiting the number of available options. We recognized that the abundance of choices could be overwhelming for newcomers. Therefore, we implemented data resampling internally, but only when the class imbalance was not hugely skewed. Our approach leveraged the pipeline functionality from scikit-learn to ensure that there was no data leakage, meaning that the test dataset remained independent of all preprocessing steps throughout the process. Additionally, autoML provided users with multiple evaluation metrics, including those capable of detecting bias introduced by resampling, such as Precision, Recall, and ROC AUC. We assumed that users would have the expertise to interpret these metrics.
However, your feedback has prompted us to reconsider the potential biases introduced by resampling, especially when users may not have the proficiency to evaluate its effects. In response to your suggestion, we have decided to remove the default resampling option altogether. Instead, we will provide users with a toggle button,

allowing them to choose whether they want to include oversampling in their workflow. We believe that this adjustment will enhance the transparency and control that users have over the training process while mitigating the risk of unintended bias. Your input has been invaluable in improving our platform, and we thank you for your constructive feedback.

---------------------------------------Reviewer's Comment
- In the context of potential datasets from different domains (including biomedical data), the datasets identified in this paper as being "large" have only up to 1500 sample and only up to 5520 features, which would not be considered large by most data scientist standards.

.........................Response to Reviewer's Comment ....
Thank you for your feedback. Indeed, a sample size of 1500 may not be considered large in today's data landscape. However, it's worth noting that in the realm of biological human samples, this dataset is one of the largest and relatively rare. Additionally, machine learning algorithms tend to perform well on larger datasets, but they can encounter challenges when dealing with smaller sample sizes or data suffering from the curse of dimensionality. Hence, we intentionally focused on these types of datasets to thoroughly test MLme's capabilities in such scenarios.
To address your concern and showcase the versatility of MLme, we have included a larger dataset comprising biometric data from 100,000 individuals, collected from the National Health Insurance Service in Korea (line 291-295). Our aim was to predict smoking status based on this extensive biometric dataset. For more detailed information about this dataset, please refer to Table S2. You can also find the corresponding in Figure S9 (A and B). We hope this addition provides a more comprehensive view of MLme's applicability across various dataset sizes and types.

---------------------------------------Reviewer's Comment
- There are largely limited details in this paper and the software's github documentation in terms of transparently indicating exactly what this pipeline does, and what options, algorithms, evaluation metrics, and visualizations it includes.

.........................Response to Reviewer's Comment ....
We appreciate your valuable feedback regarding the transparency of MLme's default pipeline and the available documentation.
We have already included the details about the default pipeline (Figure 2) used by the autoML. Furthermore, in response to your suggestion, we have taken action to enhance the clarity of MLme's functionality. Specifically, we have added an additional figure (Figure S.10, Line 235 to 237) that elucidates the list of available algorithms/steps and diverse plot types for different machine learning stages. This addition aims to provide a more comprehensive overview of the options, algorithms, evaluation metrics, and visualizations integrated into our software.

---------------------------------------Reviewer's Comment
- Since the authors do not benchmark MLme against any other autoML tool and they have a very limited set of benchmarked datasets (6 total, with limited diversity of data types, sizes, feature types), I don't think it's fair to claim that their methodology necessarily excels in it's core objective of addressing classification tasks. Ideally the authors would conduct benchmarking comparisons to STREAMLINE, as well as other autoML toolkits, however this might also understandably be outside the scope of this current paper. I do suggest the authors be more conservative in what assertions they make and conclusions they draw with respect to MLme. The authors might consider using established ML or AutoML benchmark benchmark datasets used by other algorithms and frameworks to compare or facilitate comparison of their pipeline toolkit to others.

.........................Response to Reviewer's Comment ....
Thank you for your valuable input. Here, it's important to clarify that MLme doesn't

	introduce a new methodology; rather, it leverages established ML algorithms like SVM and KNN at its core. As a result, we anticipate similar performance to other automatic ML tools.<br>To address your concern, we have conducted a comparison between MLme, TPOT and hyperopt-sklearn, and as expected, the results from both tools demonstrate comparability. For specific details and results, please refer to lines 307-315 and Figure S9.C . We believe this additional information will provide valuable insights to our readers.
Additional Information:	
Question	Response
Are you submitting this manuscript to a special series or article collection?	No
**Experimental design and statistics**<br><br>Full details of the experimental design and statistical methods used should be given in the Methods section, as detailed in our Minimum Standards Reporting Checklist. Information essential to interpreting the data presented should be made available in the figure legends.<br><br>Have you included all the information requested in your manuscript?	Yes
**Resources**<br><br>A description of all resources used, including antibodies, cell lines, animals and software tools, with enough information to allow them to be uniquely identified, should be included in the Methods section. Authors are strongly encouraged to cite Research Resource Identifiers (RRIDs) for antibodies, model organisms and tools, where possible.<br><br>Have you included the information requested as detailed in our Minimum Standards Reporting Checklist?	Yes
**Availability of data and materials**<br><br>All datasets and code on which the conclusions of the paper rely must be either included in your submission or	Yes

deposited in publicly available repositories (where available and ethically appropriate), referencing such data using a unique identifier in the references and in the "Availability of Data and Materials" section of your manuscript.<br><br>Have you have met the above requirement as detailed in our Minimum Standards Reporting Checklist?	

Manuscript
Click here to access/download;Manuscript;20230621_main_AA_ref29_9.pdf

Machine Learning Made Easy (MLme): A Comprehensive Toolkit for Machine Learning-Driven Data Analysis

Akshay Akshay[1,2,#], Mitali Katoch[3,#], Navid Shekarchizadeh[4,5], Masoud Abedi[4], Ankush Sharma[6,7], Fiona C. Burkhard[1,8], Rosalyn M. Adam[9,10,11], Katia Monastyrskaya[1, 8] and Ali Hashemi Gheinani[1,8,9,10,11*]

[1] Functional Urology Research Group, Department for BioMedical Research DBMR, University of Bern, Switzerland

[2] Graduate School for Cellular and Biomedical Sciences, University of Bern, Switzerland

[3] Institute of Neuropathology, Universitätsklinikum Erlangen, Friedrich-Alexander-Universität Erlangen-Nürnberg (FAU), Erlangen, Germany

[4] Department of Medical Data Science, Leipzig University Medical Centre, 04107 Leipzig, Germany

[5] Center for Scalable Data Analytics and Artificial Intelligence (ScaDS.AI) Dresden/Leipzig, 04105 Leipzig, Germany

[6] KG Jebsen Centre for B-cell malignancies, Institute for Clinical Medicine, University of Oslo, Oslo, Norway

[7] Department of Cancer Immunology, Institute for Cancer Research, Oslo University Hospital, Oslo, Norway

[8] Department of Urology, Inselspital University Hospital, 3010 Bern, Switzerland

[9] Urological Diseases Research Center, Boston Children's Hospital, MA, USA

[10] Harvard Medical School, Boston, Department of Surgery MA, USA

[11] Broad Institute of MIT and Harvard, Cambridge, MA, USA

[#] Contributed equally.

* Corresponding author:

Ali Hashemi Gheinani, Urological Diseases Research Center, Boston Children's Hospital, Harvard Medical School and Broad Institute of MIT and Harvard, Cambridge, MA, USA

e-mail: Ali.HashemiGheinani@childrens.harvard.edu

Keywords

- Machine learning
- Classification problems
- Data analysis
- AutoML
- Visualization

Key Points

- MLme is a novel tool that simplifies machine learning (ML) for researchers by integrating Data Exploration, AutoML, CustomML, and Visualization functionalities.

- MLme improves efficiency and productivity by streamlining the ML workflow and eliminating the need for extensive coding efforts.

- Rigorous testing on diverse datasets demonstrates MLme's promising performance in classification problems.

- MLme provides intuitive interfaces for data exploration, automated ML, customizable ML pipelines, and result visualization.

- Future developments aim to expand MLme's capabilities to include support for unsupervised learning, regression, hyperparameter tuning, and integration of user-defined algorithms.

Abstract

Background

Machine learning (ML) has emerged as a vital asset for researchers to analyze and extract valuable information from complex datasets. However, developing an effective and robust ML pipeline can present a real challenge, demanding considerable time and effort, thereby impeding research progress. Existing tools in this landscape require a profound understanding of ML principles and programming skills. Furthermore, users are required to engage in the comprehensive configuration of their ML pipeline to obtain optimal performance.

Results

To address these challenges, we have developed a novel tool called *Machine Learning Made Easy* (MLme) that streamlines the use of ML in research, specifically focusing on classification problems at present. By integrating four essential functionalities, namely Data Exploration, AutoML, CustomML, and Visualization, MLme fulfills the diverse requirements of researchers while eliminating the need for extensive coding efforts. To demonstrate the applicability of MLme, we conducted rigorous testing on six distinct datasets, each presenting unique characteristics and challenges. Our results consistently showed promising performance across different datasets, reaffirming the versatility and effectiveness of the tool. Additionally, by utilizing MLme's feature selection functionality, we successfully identified significant markers for CD8+ naive (BACH2), CD16+ (CD16), and CD14+ (VCAN) cell populations.

Conclusion

MLme serves as a valuable resource for leveraging machine learning (ML) to facilitate insightful data analysis and enhance research outcomes, while alleviating concerns related to complex coding scripts. The source code and a detailed tutorial for MLme are available at https://github.com/FunctionalUrology/MLme.

Introduction

In the realm of research, machine learning (ML) has emerged as a vital resource for analyzing intricate datasets that conventional statistical approaches struggle to interpret[1–5]. However, the integration of machine learning (ML) into research presents a multitude of challenges. Foremost, the construction and execution of an effective ML pipeline can be daunting, requiring deep domain expertise, extensive technical knowledge, and proficient programming skills. In addition, the utilization of ML techniques demands a comprehensive understanding of the underlying principles to ensure that the trained models are unbiased and transparent.

Multiple tools have been developed to streamline the process of building and executing ML pipelines (Table S1)[6–16]. These tools often require a significant level of coding proficiency and extensive configuration to achieve optimal effectiveness. Additionally, many of these tools, serve as algorithm recommenders, functioning by running multiple ML algorithms on user-provided data and providing model performance metrics. However, this approach can limit user input and guidance, as the tools tend to prioritize automated decision-making rather than allowing users to actively participate in the process. As a result, tailoring the ML models to specific research needs and ensuring that the models align with domain knowledge and expertise can be challenging. This lack of flexibility and limited user control potentially hinders the accuracy and applicability of the research outcomes.

Machine Learning Made Easy (MLme) is a comprehensive solution aimed at bridging the gap between researchers and the inherent technical complexities of ML. It facilitates the adoption of ML techniques by simplifying the ML workflow and minimizing the typically steep learning curve associated with ML. Through its intuitive interfaces, MLme enhances accessibility and usability for researchers of varying levels of technical expertise (Figure 1).

MLme offers four important components: Data Exploration, AutoML, CustomML, and Visualization, each serving a specific purpose in understanding and extracting meaningful information from the data within the ML workflow. Through the intuitive Data Exploration feature, users easily examine their datasets and gain preliminary understanding using an interactive interface. For advanced users, the CustomML interface within MLme provides a flexible platform to design and develop tailor-made ML pipelines that align with their specific research requirements. Furthermore, it facilitates effortless interpretation and analysis of results with rich visualization capabilities.

Key features of *Machine Learning Made Easy* (MLme)

MLme is a multifaceted toolkit that equips researchers with the functionalities necessary to effectively utilize ML in their research. It consists of four distinct web interfaces, each tailored to address specific research needs, ensuring a versatile and comprehensive experience for users.

Data Exploration

The Data Exploration feature of MLme allows users to upload their datasets and explore them using a range of statistical visualizations, such as density plots, scatter matrix plots, area plots, and class distribution plots (Figure S1.A). These visualizations and statistical summaries enable users to gain a comprehensive understanding of their data, including patterns and trends within the data, data distribution, and potential outliers. A density plot, for instance, can reveal

how data is distributed, while a scatter matrix plot can identify potential correlations. Class distribution plots are particularly useful for comprehending the balance of target classes within the dataset, which can be crucial when designing a machine learning model.

Overall, the Data Exploration feature enables users to efficiently explore their datasets and acquire initial insights into their data. This knowledge can inform subsequent modeling decisions, ensuring that users are using the most appropriate modeling techniques for their specific dataset.

AutoML

The AutoML feature in MLme enables users to effortlessly extract meaningful information from their datasets using ML, even without extensive technical expertise (Figure S1.B). With a preconfigured ML pipeline (Figure 2), the AutoML handles essential preprocessing steps such as data resampling, scaling, and feature selection[17]. These steps ensure that the input data is properly prepared for ML algorithms, enhancing the performance and reliability of subsequent trained models. The AutoML conducts training and evaluation of multiple classification models, including a dummy classifier. By employing diverse models, users gain a comprehensive understanding of their data and can identify the most effective algorithms for their specific dataset.

After the pipeline is completed, the AutoML offers users various options for examining and interpreting the results. These options include intuitive and interactive plots, which help users gain a deeper understanding of the performance characteristics of the models. Additionally, users have the flexibility to download the results and explore them further using the Visualization interface at their convenience.

CustomML

The CustomML feature of MLme empowers users with moderate to advanced knowledge of the ML domain to design and customize an ML pipeline that caters to their specific research needs (Figure S2.A). With its user-friendly and intuitive interface, users can easily include or exclude steps and algorithms using a simple toggle button. This eliminates the worry about writing complex programming scripts and allows to focus on selecting the most suitable steps and algorithms for the dataset.

CustomML offers an extensive range of preprocessing options, including seven algorithms for data resampling, nineteen algorithms for scaling, and a diverse array of feature selection algorithms to select relevant features from the dataset. Moreover, with sixteen classification algorithms available, users can refine their pipeline to align with their research requirements. To provide a comprehensive understanding of the trained model's performance, CustomML supports ten different evaluation methods and fourteen evaluation metrics.

The customization options of CustomML are enhanced by allowing users to select the parameters value for all the provided algorithms, giving them greater control over the behavior of their developed pipeline. Once the pipeline is designed, it can be conveniently downloaded and executed either locally or on a cluster, offering flexibility in computing resources. The CustomML-generated ML pipeline produces a pickle file (.pkl) as an output upon completion, which contains all the results from the pipeline. This file can be uploaded to the Visualization interface, enabling users to interpret these results using various plots.

Visualization

The Visualization feature in MLme allows users to effortlessly interpret their results without the need for advanced programming skills or expertise in data visualization (Figure S2.B). It provides a comprehensive range of plots and tables, covering fundamental as well as advanced options such as bar plots, heatmaps, and spider plots. These diverse visualization tools facilitate effective comparison of trained model performance.

Furthermore, this feature allows users to customize the appearance of their plots by selecting from over fifty different color palettes. Additionally, all generated plots are of high quality and are downloadable in high resolution, ensuring they are suitable for publication purposes. Figure S10 showcases the available list of algorithms and diverse plot types within MLme for various machine learning stages.

Use-Cases

Dataset Selection Criteria

The MLme application is evaluated using seven distinct datasets (Table S2) that are carefully chosen to ensure robustness. Factors such as sample size, diversity, class imbalance, and dimensionality are considered during the selection process. The selected datasets vary in sample size and diversity, providing a comprehensive assessment of the MLme application's performance across different data scales.

This includes datasets of varying sizes, from small (chronic cymphocytic leukemia and cervical cancer study) to large (invasive breast carcinoma and body signal datasets), which test the application's scalability and efficiency. Imbalanced datasets, like invasive Breast Carcinoma (BRCA), are included to evaluate the MLme application's handling of class imbalance and prediction accuracy, which is particularly relevant in real-world scenarios, such as biological research. The datasets also address the challenge of high-dimensional features and low sample sizes, known as the curse of dimensionality. By including such datasets, the MLme ability to handle challenges is thoroughly assessed.

Furthermore, the Glass Identification dataset was selected as a non-biological example, offering variation, and enabling testing across diverse domains. This dataset, with multiple target classes, allows evaluation of the MLme application's performance in multi-class classification problems.

Dataset Descriptions

The first dataset comprised of mRNA patient data (n=136) obtained from a study on Chronic Lymphocytic Leukemia (CLL), which measured their transcriptome profiles[18]. Our objective was to build a model that could classify male and female patients based on their transcriptomic profiles, using the top 5,000 most variable mRNAs (excluding Y chromosome genes). The second dataset was collected from a cervical cancer study that analyzed the expression levels of 714 miRNAs in human samples (n=58)[19].

The third and fourth datasets were obtained from The Cancer Genome Atlas (TCGA), consisting of mRNA (n=1219) and miRNA (n=1207) sequencing data from patients with

invasive BRCA, which were retrieved using the TCGAbiolinks package[20] in *R*. For the BRCA mRNA dataset, we focused only on differentially expressed genes from edgeR (FDR ≤ 0.001 and logFC > ±2)[21]. Our goal was to train a model capable of distinguishing normal and tumor samples for both cervical cancer and TCGA-BRCA datasets.

The fifth dataset consists of scRNA-seq data obtained from peripheral blood mononuclear cells (PBMCs) that were sequenced using 10× chromium technology[22]. Among all the cell populations described in this study, we specifically utilized the scRNA datasets of CD8+ naive, CD14+, and CD16+ monocytes (n=1500) with the goal of identifying distinct markers for each of these cell populations.

The sixth dataset utilized in this study was the widely recognized Glass Identification dataset (n=214) obtained from the University of California Irvine (UCI) ML repository[23]. This dataset comprises 10 distinct features that represents oxide content of glass samples. The primary objective of this dataset is to classify different types of glass based on their oxide content.

The seventh dataset in our study comprises body signal data collected from 100,000 individuals through the National Health Insurance Service in Korea[24]. This dataset includes 21 essential biological signals related to health, such as measurements of systolic blood pressure and total cholesterol levels. Our main goal with this dataset was to determine whether individuals consume alcohol based on the available biological signal information.

Results

To perform a thorough assessment of the MLme functionality, we utilized its customML feature to construct distinct ML pipelines for CLL, Cervical cancer, Body signal and TCGA datasets. These pipelines entailed various processing steps, including data scaling and resampling using different algorithms, multiple ML classifiers, diverse evaluation methods, and metrics. Additionally, we employed the AutoML feature of MLme to train multiple models for both the PBMC and glass datasets. The top-performing models consistently achieved scores exceeding 90% for all computed metrics across all evaluated datasets except Glass Identification and Body signal dataset. As anticipated, the dummy classifiers performed the worst among all the datasets (Figures S3-S9). Additionally, we conducted a comparative analysis to assess the performance of MLme in comparison to TPOT and hyperopt-sklearn on these datasets. The fact that all three tools demonstrated similar performance (Figure S9.C) for all datasets, except the glass dataset, underscores the reliability and consistency of the results produced by MLme. For Hyperopt-sklearn, we configured it to comprehensively explore all classification algorithms and data transformations within the library while utilizing the Tree-structured Parzen Estimator (TPE) algorithm for hyperparameter search. For TPOT, we employed a five-minute runtime limit, a population size of 50, five generations, and default values for all other parameters.

To further demonstrate the applicability of MLme, we utilized its feature selection functionality from AutoML to identify the most important genes for classifying CD8+ naive, CD14+, and CD16+ monocyte cell populations from the PBMC dataset. By selecting the top 10% of the original input of 500 highly variable genes, MLme provided a list of 50 genes that are sufficient for classifying these cell types (Figure 3A). These 50 genes exhibited a strong correspondence with their respective cell populations, except for 13 ribosomal genes (RPS and RPL) that showed similar expression levels across all three cell types.

Among the remaining 37 genes, we discovered classic markers for CD8+ naive cells (TCF7[25,26], LEF1[25], BACH2[27], BCL11B[28], and THEMIS[29]), which have been previously described in the literature (Figure 3B). The list also included markers for the CD16+ cell population, such as FCGR3A (CD16), TCF7L2, MS4A7, IFITM3, MTSS1, LST1, and WARS (Figure 3C), which have been associated with CD16+ cells in previous studies[30,31]. Furthermore, our marker list encompassed known CD14+ specific genes, including VCAN, a marker of monocytic lineage[32], CSF3R, previously described in the CD14+ population[33], and NEAT1 (Figure 3D). These findings validate the biological relevance of the selected genes and highlight the utility of the MLme tool in biomedical research.

Implementation

The MLme is developed using *Dash* library[34] in the *Python*[35] programming language. Plots are generated using *Plotly*[36], *matplotlib*[37], and *bokeh*[38] libraries. *Pandas*[39] and *NumPy*[40] libraries are used to handle data storage and processing. The development of the ML pipeline is facilitated by employing the *Scikit-Learn*[41] and *Imbalanced Learn*[42] libraries.

Limitations

Currently, MLme focuses on classification problems since a substantial portion of research questions and available datasets are aligned with the domain of classification. This limitation hinders MLme applicability to regression or unsupervised learning tasks. Additionally, the tool lacks built-in hyperparameter tuning capabilities. This absence of a key feature may hinder users in fine-tuning their models.

Overall, while the current version of the MLme has limitations concerning regression and unsupervised learning problems, it excels in its core objective of addressing classification tasks. It is worth noting that users have the flexibility to choose values for all the parameters of a given algorithm through the user interface, to some extent mitigating the impact of the lack of built-in hyperparameter tuning to some extent.

Conclusion

Our paper introduces a user-friendly tool called MLme, which offers a wide range of functionalities for ML analysis. Its primary goal is to make machine learning (ML) accessible to users of all skill levels by removing technical barriers. With the Data Exploration feature, users can efficiently explore datasets and gain initial insights into their data. The AutoML feature simplifies ML usage, allowing them to leverage ML capabilities without dealing with complex technicalities. Moreover, the CustomML functionality assists in creating personalized pipelines using an intuitive graphical user interface that caters to specific requirements, eliminating the need for coding complexities. Additionally, the visualization features enable users to interactively explore and understand model performance, without extensive data visualization or coding expertise. In summary, MLme is a powerful and user-friendly tool that empowers researchers to enhance their research outcomes through ML.

However, it is crucial to emphasize that, despite their impressive capabilities, automated ML tools should never be regarded as a replacement for domain expertise. Users of MLme must maintain a strong awareness of the invaluable role that domain knowledge plays when using

this software to address real-world problems. Consequently, expertise in the specific field remains irreplaceable, and MLme should be viewed as a complementary tool to augment, rather than replacement, human understanding and insights.

Outlook

Despite the limitations mentioned above, there are several promising directions for future development of the MLme. Our primary objective is to expand the capabilities of MLme to include support for unsupervised learning and regression problems. This expansion will greatly enhance the tool's utility and enable its application in a broader range of ML tasks.

Recognizing the importance of hyperparameter tuning in optimizing models, we plan to incorporate hyperparameter tuning capabilities into the tool. This addition will enable users to fine-tune their models and improve overall performance, thereby increasing the MLme effectiveness and reliability. Additionally, we intend to introduce a feature that allows users to upload and integrate their own algorithms into the pipeline. This feature will enable users to use their preferred algorithms, even if they are not currently available within the tool, thereby expanding its applicability and customization options.

These future developments aim to overcome the current limitations of MLme and enhance its functionality and adaptability. By addressing these limitations, we firmly believe that the MLme will evolve into a more comprehensive and valuable resource for ML practitioners.

Availability of supporting source code and requirements

Project name: Machine Learning Made Easy (MLme)

Project home page: https://github.com/FunctionalUrology/MLme

BioTool ID: MLme

SciCrunch ID: MLme (RRID: SCR_024439)

Operating system(s): Platform independent

Programming language: Python (version 3.9)

Other requirements: Docker or Python

License: GNU GPL

Author contributions statement

K.M., A.H.G, A.A., and M.K. conceived the idea for the manuscript. A.A. and M.K. wrote the source code and conducted testing and debugging of the MLme. K.M., F.C.B, R.M.A, A.H.G, and A.S. provided feedback on the biological application of the tool. N.S., M.A., A.H.G, and A.S. provided technical feedback throughout the development phase and participated in testing and debugging. K.M., A.S. and M.K. wrote the manuscript with inputs from all the other authors. All authors contributed to proofreading and revising the manuscript.

Funding

We gratefully acknowledge the financial support of the Swiss National Science Foundation (SNF Grant 310030_175773 to FCB and KM, 212298 to FCB and AHG) and the Wings for Life Spinal Cord Research Foundation (WFL-AT-06/19 to KM). AHG and RMA are supported by R01 DK127673. MK is supported by the Else Kröner-Fresenius-Stiftung (EKFS 2021_EKeA.33). The authors acknowledge the financial support from the Federal Ministry of Education and Research of Germany and by the Sächsische Staatsministerium für Wissenschaft Kultur und Tourismus in the program Center of Excellence for AI-research "Center for Scalable Data Analytics and Artificial Intelligence Dresden/Leipzig" (project identification number: ScaDS.AI).

Conflict of Interest

The authors have declared no competing interests.

Data Availability

All supporting data, including the input dataset, 'inputParameters.pkl', and 'results.pkl' files, for all evaluated datasets, is available on Zenodo[43]. The 'results.pkl' files can be visualized using the Visualization feature of MLme. DOME-ML (Data, Optimisation, Model, and Evaluation in Machine Learning) annotation, supporting the current study, is available through DOME Wizard.

Acknowledgment

We would like to express our sincere gratitude to Pedro Perreira Amado for his invaluable contribution in testing MLme.

Reference:

1. Lewis, J. E. & Kemp, M. L. Integration of machine learning and genome-scale metabolic modeling identifies multi-omics biomarkers for radiation resistance. *Nat Commun* **12**, 2700 (2021).
2. Tollenaar, V. *et al.* Unexplored Antarctic meteorite collection sites revealed through machine learning. *Science Advances* **8**, eabj8138 (2022).
3. Su, Q. *et al.* Faecal microbiome-based machine learning for multi-class disease diagnosis. *Nat Commun* **13**, 6818 (2022).
4. Martínez, B. A. *et al.* Machine learning reveals distinct gene signature profiles in lesional and nonlesional regions of inflammatory skin diseases. *Science Advances* **8**, eabn4776 (2022).
5. Chen, Z. *et al.* Using machine learning to estimate the incidence rate of intimate partner violence. *Sci Rep* **13**, 5533 (2023).
6. Hall, M. *et al.* The WEKA data mining software: an update. *SIGKDD Explor. Newsl.* **11**, 10–18 (2009).
7. Thornton, C., Hutter, F., Hoos, H. H. & Leyton-Brown, K. Auto-WEKA: combined selection and hyperparameter optimization of classification algorithms. in *Proceedings of the 19th ACM SIGKDD international conference on Knowledge discovery and data mining* 847–855 (Association for Computing Machinery, 2013). doi:10.1145/2487575.2487629.
8. Frank, E., Hall, M. A. & Witten, I. H. *The WEKA Workbench. Data Mining: Practical Machine Learning Tools and Techniques* (Morgan Kaufmann, 2016).
9. Salesforce. Transmogrifai. https://docs.transmogrif.ai/en/stable/ (2019).
10. Urbanowicz, R., Zhang, R., Cui, Y. & Suri, P. STREAMLINE: A Simple, Transparent, End-To-End Automated Machine Learning Pipeline Facilitating Data Analysis and

Algorithm Comparison. in *Genetic Programming Theory and Practice XIX* (eds. Trujillo, L., Winkler, S. M., Silva, S. & Banzhaf, W.) 201–231 (Springer Nature, 2023). doi:10.1007/978-981-19-8460-0_9.

11. Axel. AxeldeRomblay/MLBox. (2023).
12. Jin, H., Chollet, F., Song, Q. & Hu, X. AutoKeras: An AutoML Library for Deep Learning. *Journal of Machine Learning Research* **24**, 1–6 (2023).
13. Komer, B., Bergstra, J. & Eliasmith, C. Hyperopt-Sklearn. in *Automated Machine Learning: Methods, Systems, Challenges* (eds. Hutter, F., Kotthoff, L. & Vanschoren, J.) 97–111 (Springer International Publishing, 2019). doi:10.1007/978-3-030-05318-5_5.
14. Le, T. T., Fu, W. & Moore, J. H. Scaling tree-based automated machine learning to biomedical big data with a feature set selector. *Bioinformatics* **36**, 250–256 (2020).
15. La Cava, W. *et al.* Evaluating recommender systems for AI-driven biomedical informatics. *Bioinformatics* **37**, 250–256 (2021).
16. Feurer, M., Eggensperger, K., Falkner, S., Lindauer, M. & Hutter, F. Auto-sklearn 2.0: hands-free AutoML via meta-learning. *J. Mach. Learn. Res.* **23**, 261:11936-261:11996 (2022).
17. Akshay, A. MLme: Machine Learning Made Easy. (2023) doi:10.48546/WORKFLOWHUB.WORKFLOW.571.1.
18. Dietrich, S. *et al.* Drug-perturbation-based stratification of blood cancer. *J Clin Invest* **128**, 427–445 (2018).
19. Witten, D., Tibshirani, R., Gu, S. G., Fire, A. & Lui, W.-O. Ultra-high throughput sequencing-based small RNA discovery and discrete statistical biomarker analysis in a collection of cervical tumours and matched controls. *BMC Biology* **8**, 58 (2010).
20. Colaprico, A. *et al.* TCGAbiolinks: an R/Bioconductor package for integrative analysis of TCGA data. *Nucleic Acids Research* **44**, e71 (2016).

21. Robinson, M. D., McCarthy, D. J. & Smyth, G. K. edgeR: a Bioconductor package for differential expression analysis of digital gene expression data. *Bioinformatics* **26**, 139–140 (2010).
22. Home Page. *10x Genomics* https://www.10xgenomics.com/.
23. Dua, D. & Graff, C. UCI Machine Learning Repository. (2017).
24. Her, S. Smoking and Drinking Dataset with body signal. *Kaggle* https://www.kaggle.com/datasets/sooyoungher/smoking-drinking-dataset.
25. Xing, S. *et al.* Tcf1 and Lef1 transcription factors establish CD8+ T cell identity through intrinsic HDAC activity. *Nat Immunol* **17**, 695–703 (2016).
26. Zhang, J., Lyu, T., Cao, Y. & Feng, H. Role of TCF-1 in differentiation, exhaustion, and memory of CD8+ T cells: A review. *The FASEB Journal* **35**, e21549 (2021).
27. Roychoudhuri, R. *et al.* BACH2 regulates CD8+ T cell differentiation by controlling access of AP-1 factors to enhancers. *Nat Immunol* **17**, 851–860 (2016).
28. Helm, E. Y. *et al.* Bcl11b sustains multipotency and restricts effector programs of intestinal-resident memory CD8+ T cells. *Science Immunology* **8**, eabn0484 (2023).
29. Tang, J. *et al.* Themis suppresses the effector function of CD8+ T cells in acute viral infection. *Cell Mol Immunol* **20**, 512–524 (2023).
30. Ancuta, P. *et al.* Transcriptional profiling reveals developmental relationship and distinct biological functions of CD16+ and CD16- monocyte subsets. *BMC Genomics* **10**, 403 (2009).
31. Hu, Y. *et al.* Genetic landscape and autoimmunity of monocytes in developing Vogt–Koyanagi–Harada disease. *Proc. Natl. Acad. Sci. U.S.A.* **117**, 25712–25721 (2020).
32. Affandi, A. J. *et al.* CD169 Defines Activated CD14+ Monocytes With Enhanced CD8+ T Cell Activation Capacity. *Frontiers in Immunology* **12**, (2021).

33. Combes, T. W. *et al.* CSF1R defines the mononuclear phagocyte system lineage in human blood in health and COVID-19. *Immunother Adv* **1**, ltab003 (2021).
34. Hossain, S. Visualization of Bioinformatics Data with Dash Bio. *Proceedings of the 18th Python in Science Conference* 126–133 (2019) doi:10.25080/Majora-7ddc1dd1-012.
35. van Rossum, G. Python reference manual. (1995).
36. Inc, P. T. Collaborative data science. https://plot.ly (2015).
37. Hunter, J. D. Matplotlib: A 2D Graphics Environment. *Computing in Science & Engineering* **9**, 90–95 (2007).
38. Bokeh Development Team. *Bokeh: Python library for interactive visualization*. (2018).
39. McKinney, W. Data Structures for Statistical Computing in Python. *Proceedings of the 9th Python in Science Conference* 56–61 (2010) doi:10.25080/Majora-92bf1922-00a.
40. Harris, C. R. *et al.* Array programming with NumPy. *Nature* **585**, 357–362 (2020).
41. Pedregosa, F. *et al.* Scikit-learn: Machine Learning in Python. *Journal of Machine Learning Research* **12**, 2825–2830 (2011).
42. Lemaıtre, G. & Nogueira, F. Imbalanced-learn: A Python Toolbox to Tackle the Curse of Imbalanced Datasets in Machine Learning.
43. Akshay, A. *et al.* Supporting data for ‘Machine Learning Made Easy (MLme): A Comprehensive Toolkit for Machine Learning-Driven Data Analysis’. (2023) doi:10.5281/zenodo.8073635.

Figure
Click here to access/download;Figure;figures29_9.pdf

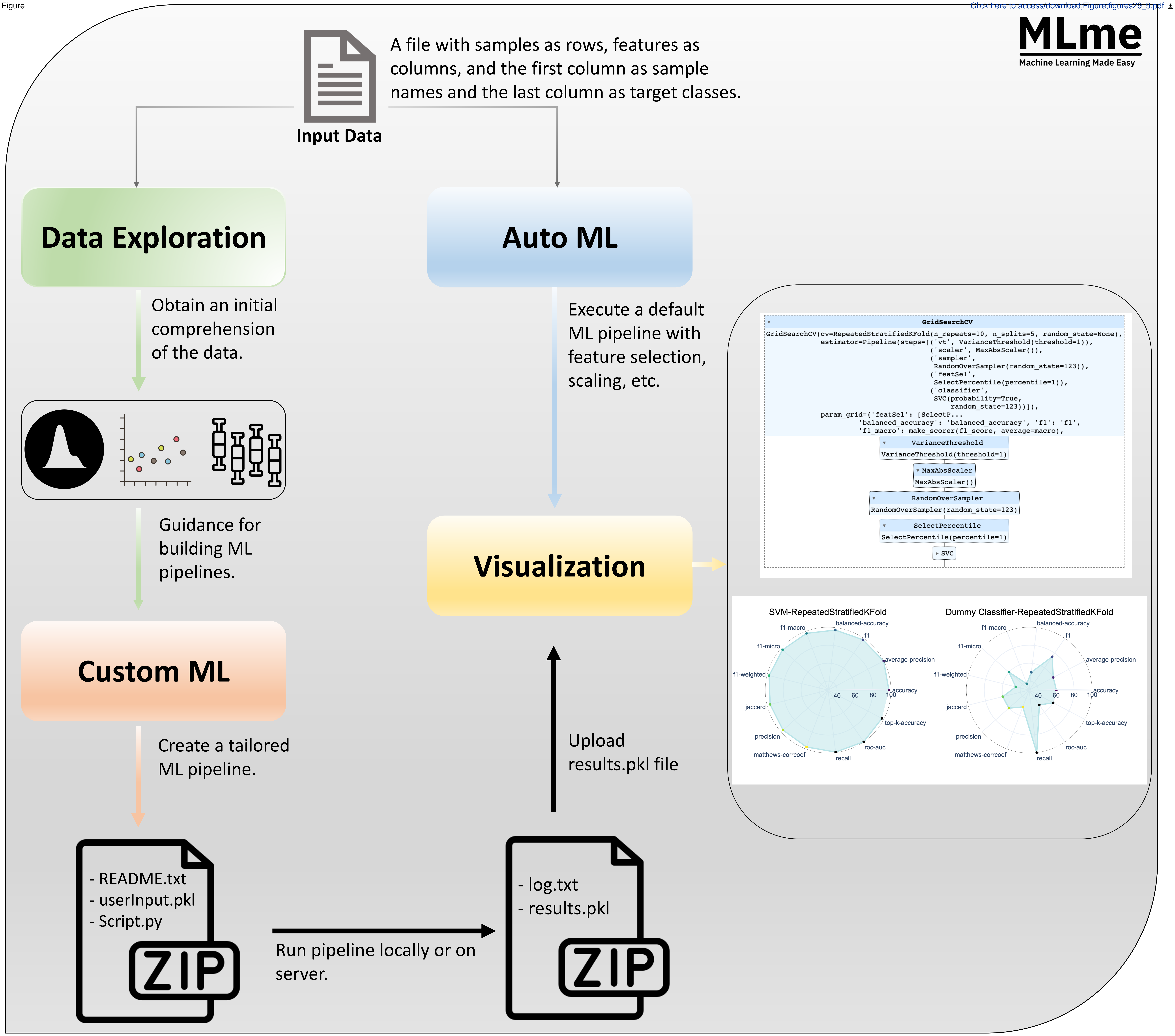

Figure 1. Graphical abstract. The input data for Machine Learning Made Easy (MLme) is a file with samples as rows and features as columns, with sample names in the first column and target classes in the last column. MLme provides various features to enhance usability. The data exploration feature enables users to explore the data and gain initial insights. For advanced users, the custom ML feature allows the creation of custom ML pipelines. Upon execution, MLme generates a compressed zip file containing inputParameter.pkl, script.py, and README.txt. Alternatively, users can opt for the AutoML feature, which applies a default ML pipeline to the input file. Both customML and AutoML produce a results.pkl file, which can be further analyzed using the visualization feature.

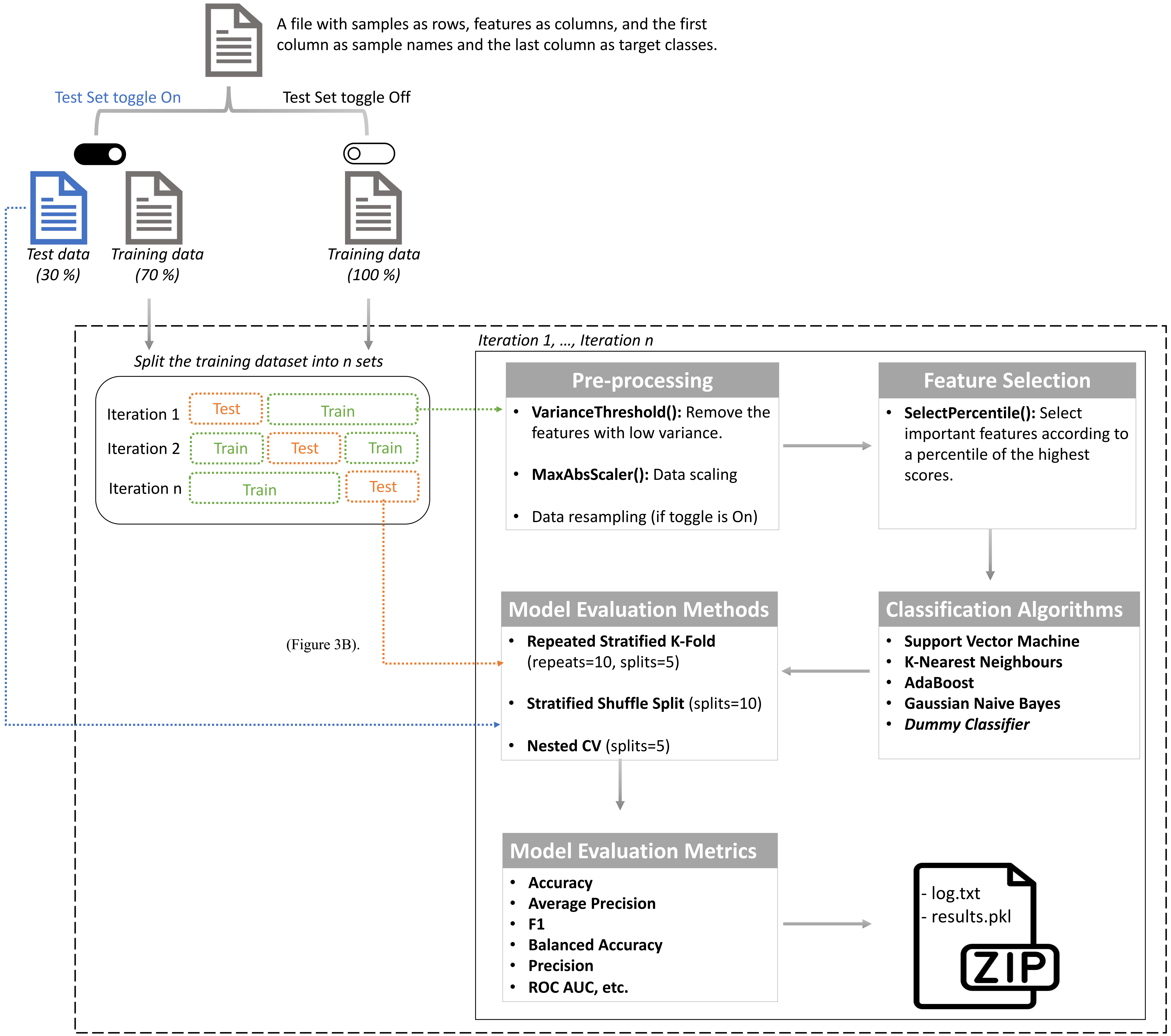

Figure 2. Default ML Pipeline for AutoML. The default ML pipeline can be represented as a flowchart that starts by splitting the input dataset into training and independent test sets, provided the user has activated the test set option. Otherwise, the entire dataset is used for training. In the subsequent step, the training dataset is divided into n bins of equal size through stratified sampling. From these bins, k-1 are designated as training sets while the remainder becomes the test set. In the pre-processing step, low variance features are removed first, followed by data scaling and resampling. Subsequently, the SelectPercentile univariate feature selection method is applied to select important features, and five ML classification algorithms are trained. Model performance is assessed on the test set using three different methods, and multiple performance metrics are computed. This entire process is repeated for each unique bin in the k-fold CV method. The pipeline outputs a zip file comprising the log .txt and the results.pkl files. The user can examine the results by visualizing the contents of the pickle file using Machine Learning Made Easy (MLme).

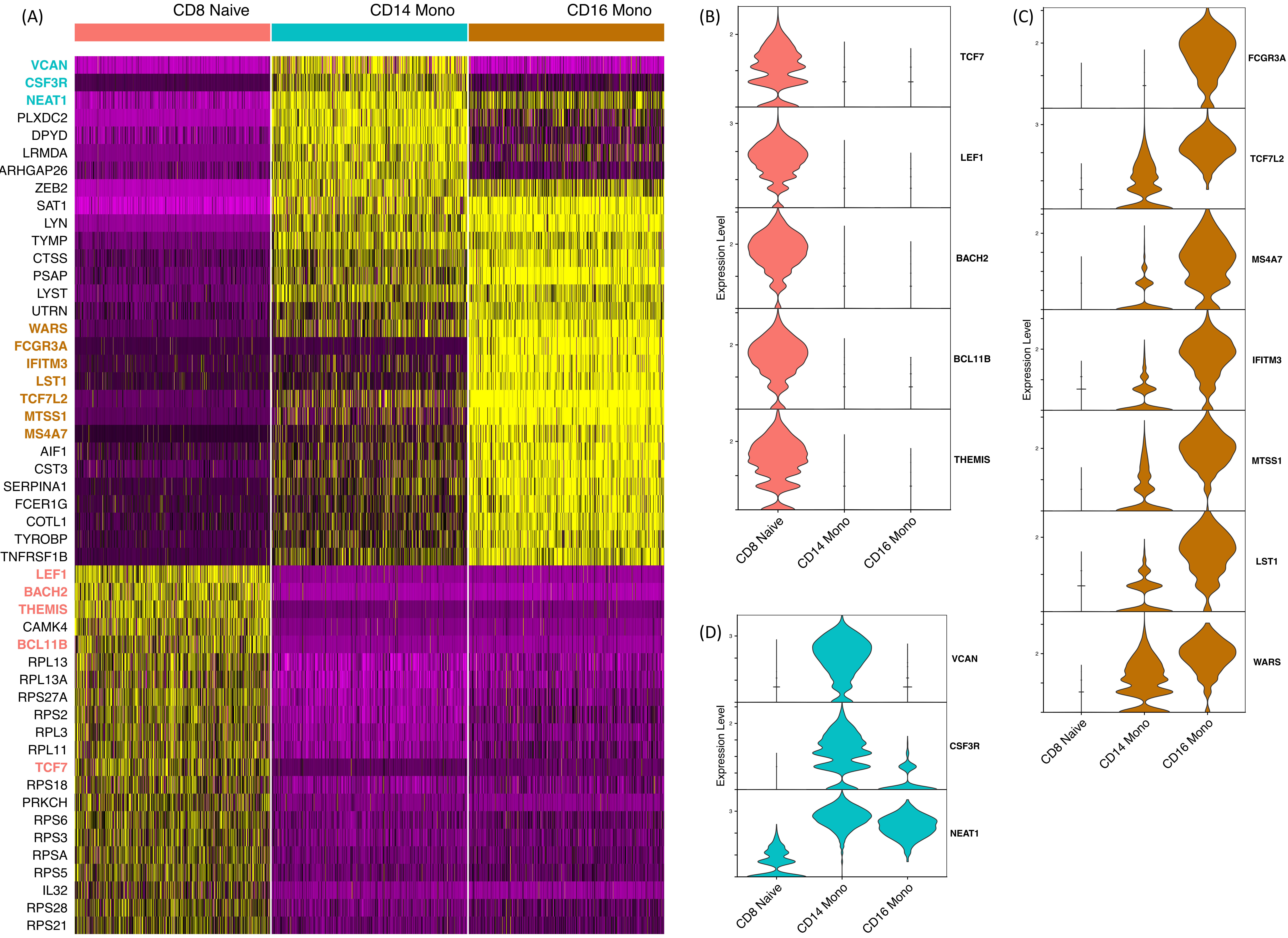

Figure 3. Identification of Potential Markers for CD8+ Naïve, CD16+, and CD14+ Cell Populations in PBMC Dataset. (A) Heatmap visualization showing the expression patterns of 50 genes selected by MLme. **(B), (C), and (D)** Demonstrate the expression levels of key markers specific to CD8+ naïve, CD16+, and CD14+ cell populations, respectively, within each cell type.

Click here to access/download
Supplementary Material
figure-suppl29_9.pdf

Click here to access/download
Supplementary Material
supplementary29_9.pdf
