# Supplementary material for: Machine Learning Made Easy (MLme): a comprehensive toolkit for machine learning–driven data analysis
Source: Gigascience. 2024 Jan 11;13:giad111. doi: 10.1093/gigascience/giad111 (PMC10783149; doi:10.1093/gigascience/giad111)
Supplement: giad111_Supplemental_Files [file giad111_supplemental_files.zip › figure-suppl_rev_3.pdf]

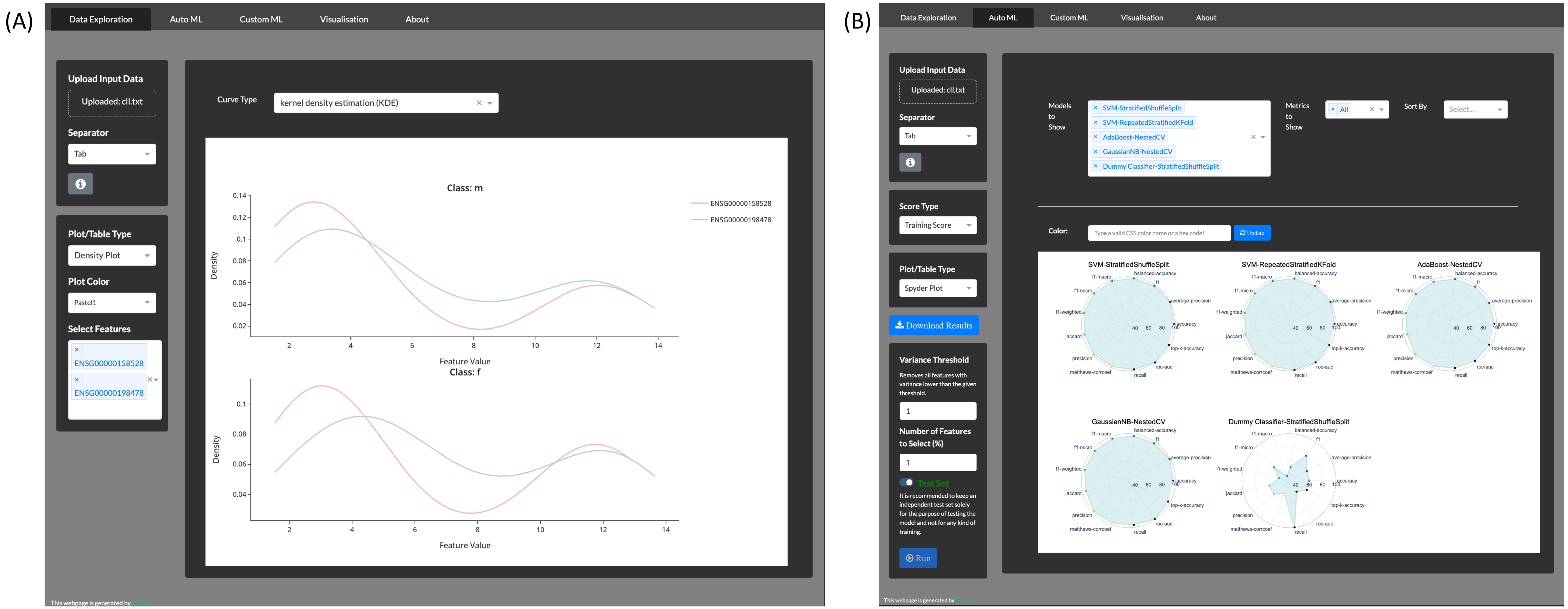

Figure S1. Key features of Machine Learning Made Easy (MLme). (A) Data Exploration, **(B)** AutoML.

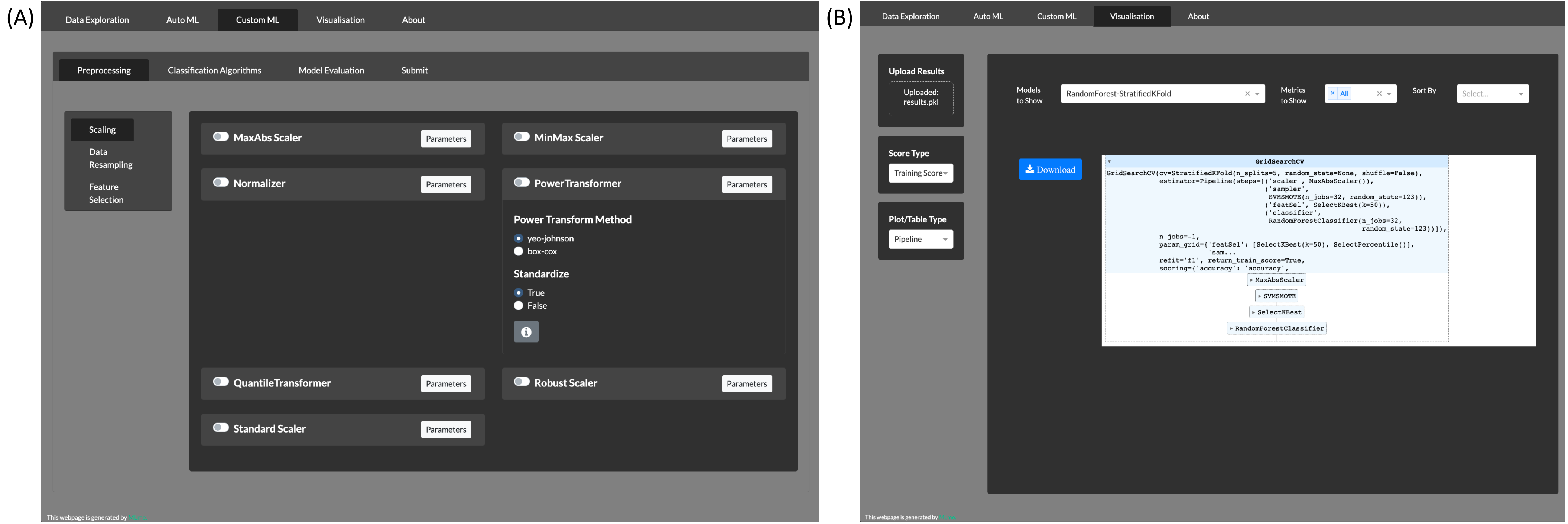

Figure S2. Key features of Machine Learning Made Easy (MLme). (A) CustomML, **(B)** Visualization.

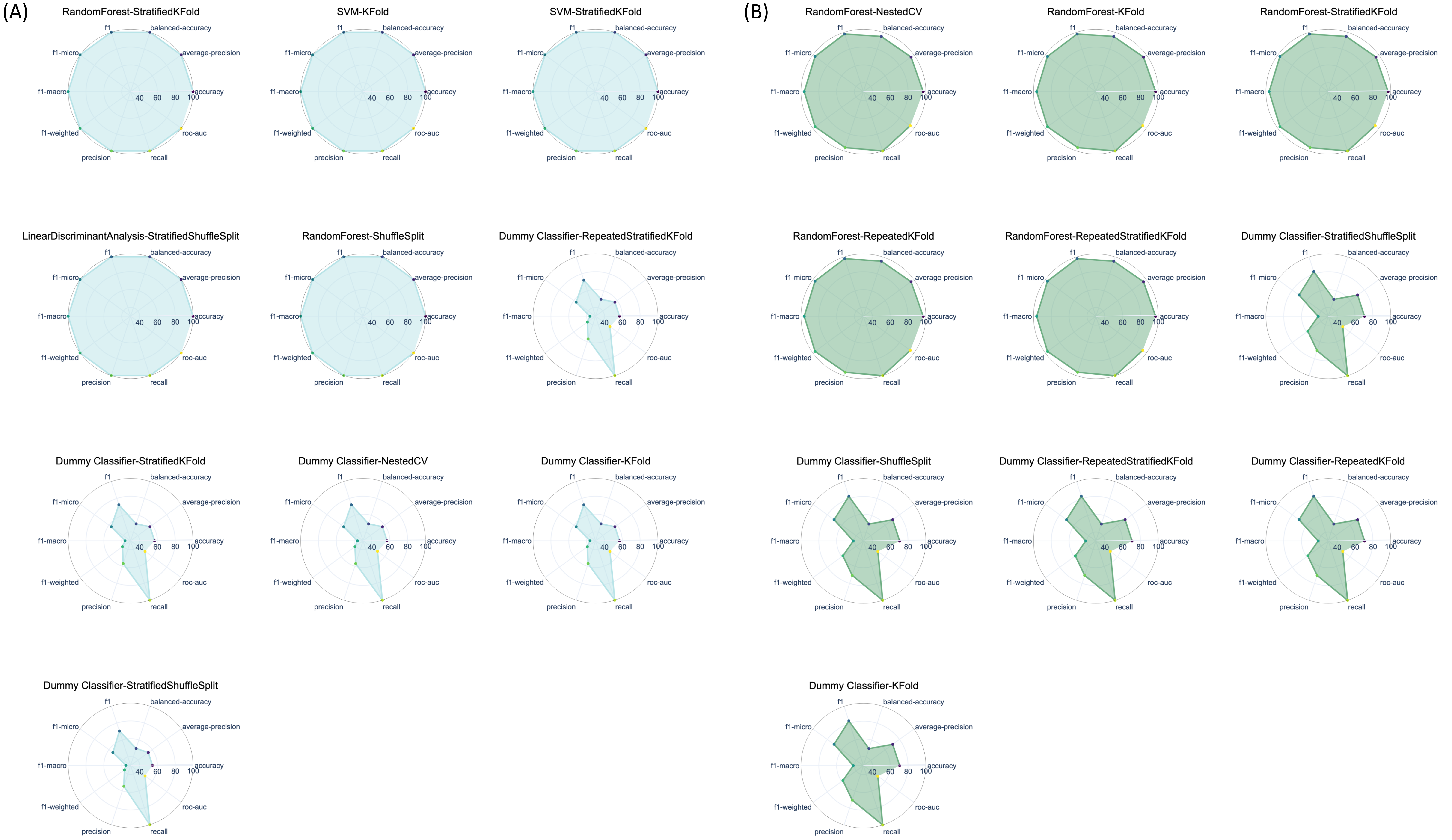

Figure S3. Projection of metrics scores on two-dimensional (2D) polar coordinates. The plots illustrate the performance scores of the top and worst five machine learning (ML) algorithms trained on the Chronic Lymphocytic Leukemia (CLL) dataset, both during training (A) and testing (B). Each ML model is represented by a circle, and each vertex represents a specific performance metric. A circle with a larger shaded area indicates better performance.

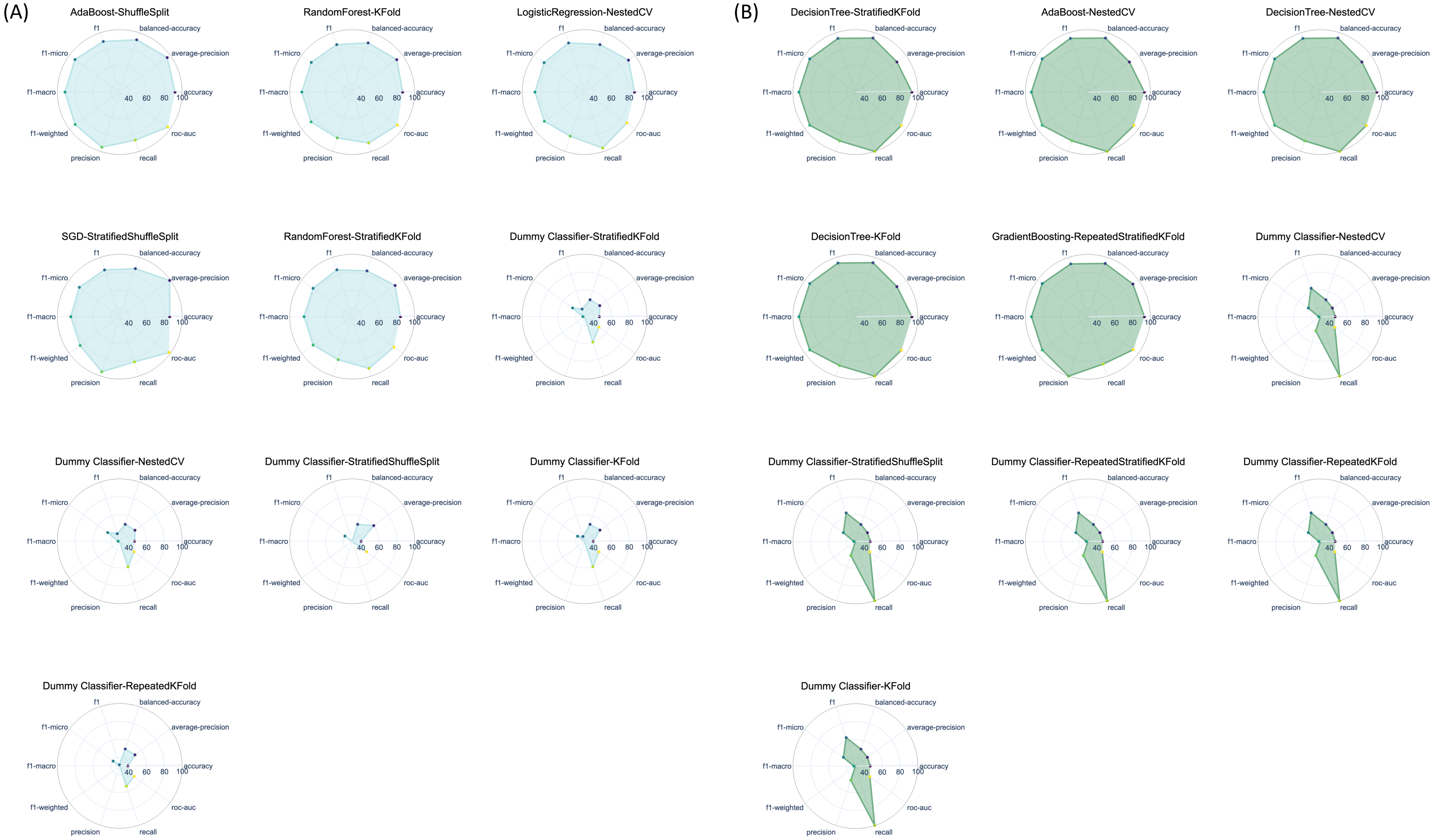

Figure S4. Projection of metrics scores on two-dimensional (2D) polar coordinates. The plots illustrate the performance scores of the top and worst five machine learning (ML) algorithms trained on the Cervical Cancer dataset, both during training (A) and testing (B). Each ML model is represented by a circle, and each vertex represents a specific performance metric. A circle with a larger shaded area indicates better performance.

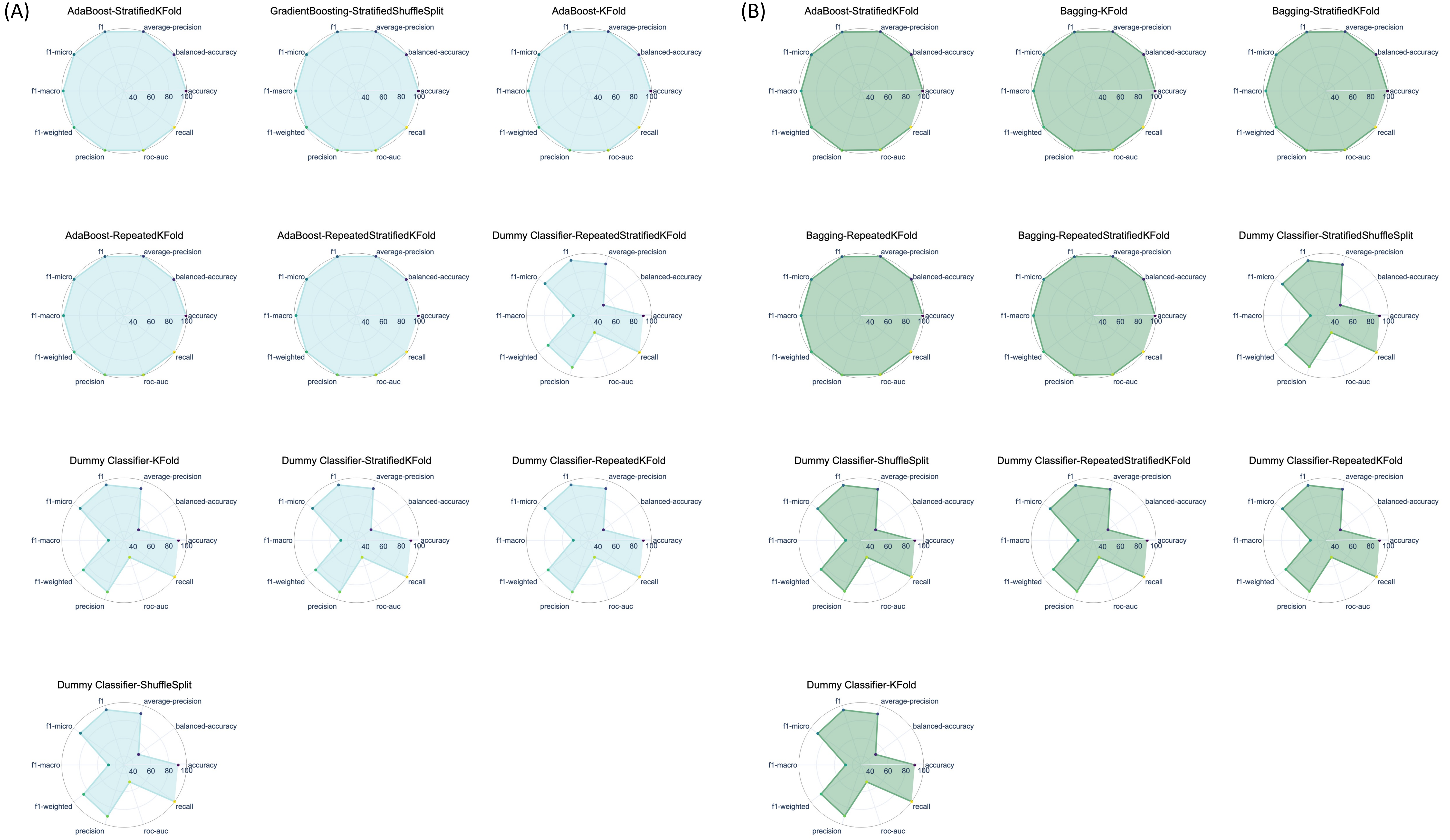

Figure S5. Projection of metrics scores on two-dimensional (2D) polar coordinates. The plots illustrate the performance scores of the top and worst five machine learning (ML) algorithms trained on the TCGA mRNA dataset, both during training (A) and testing (B). Each ML model is represented by a circle, and each vertex represents a specific performance metric. A circle with a larger shaded area indicates better performance.

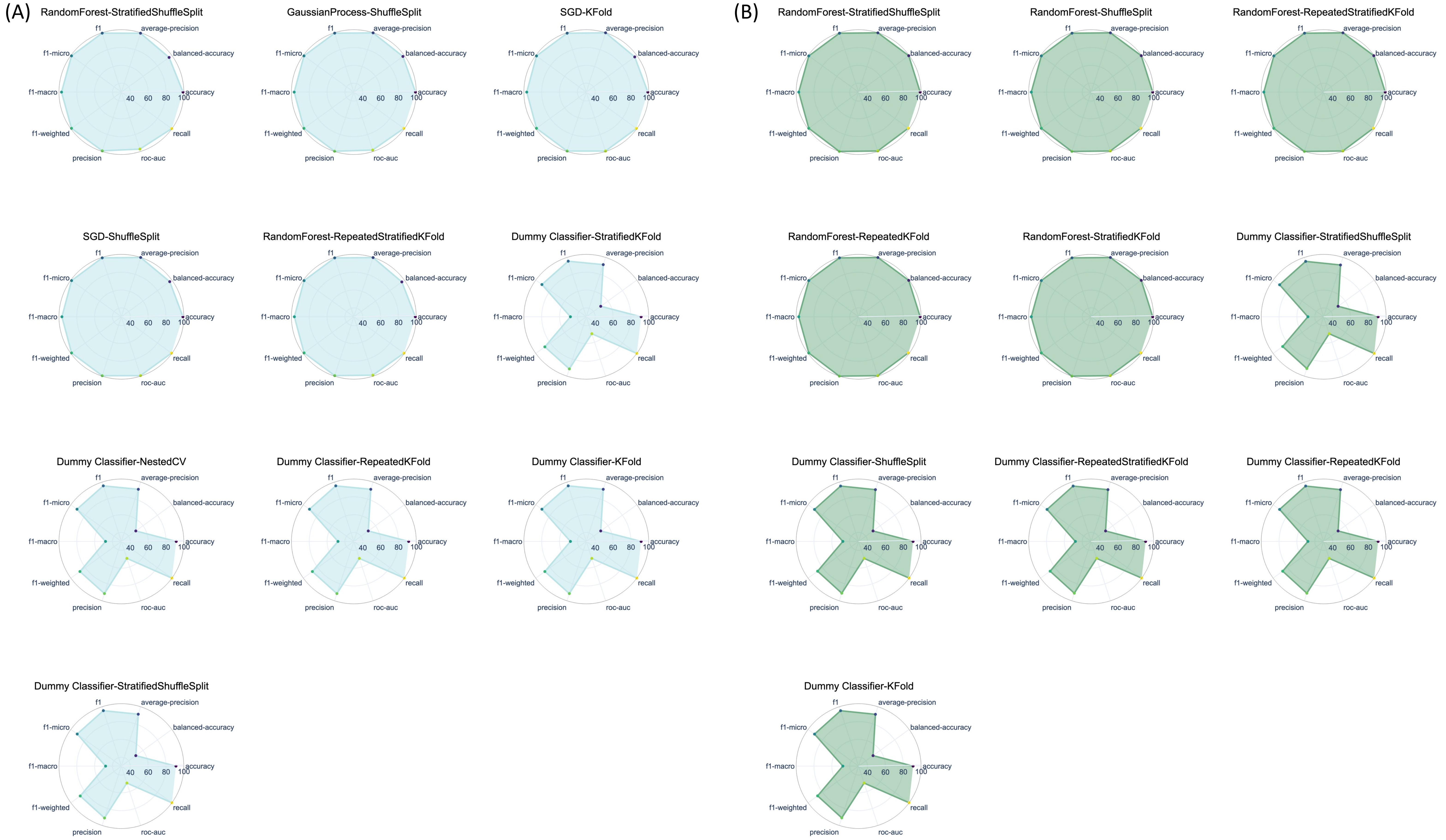

Figure S6. Projection of metrics scores on two-dimensional (2D) polar coordinates. The plots illustrate the performance scores of the top and worst five machine learning (ML) algorithms trained on the TCGA miRNA dataset, both during training (A) and testing (B). Each ML model is represented by a circle, and each vertex represents a specific performance metric. A circle with a larger shaded area indicates better performance.

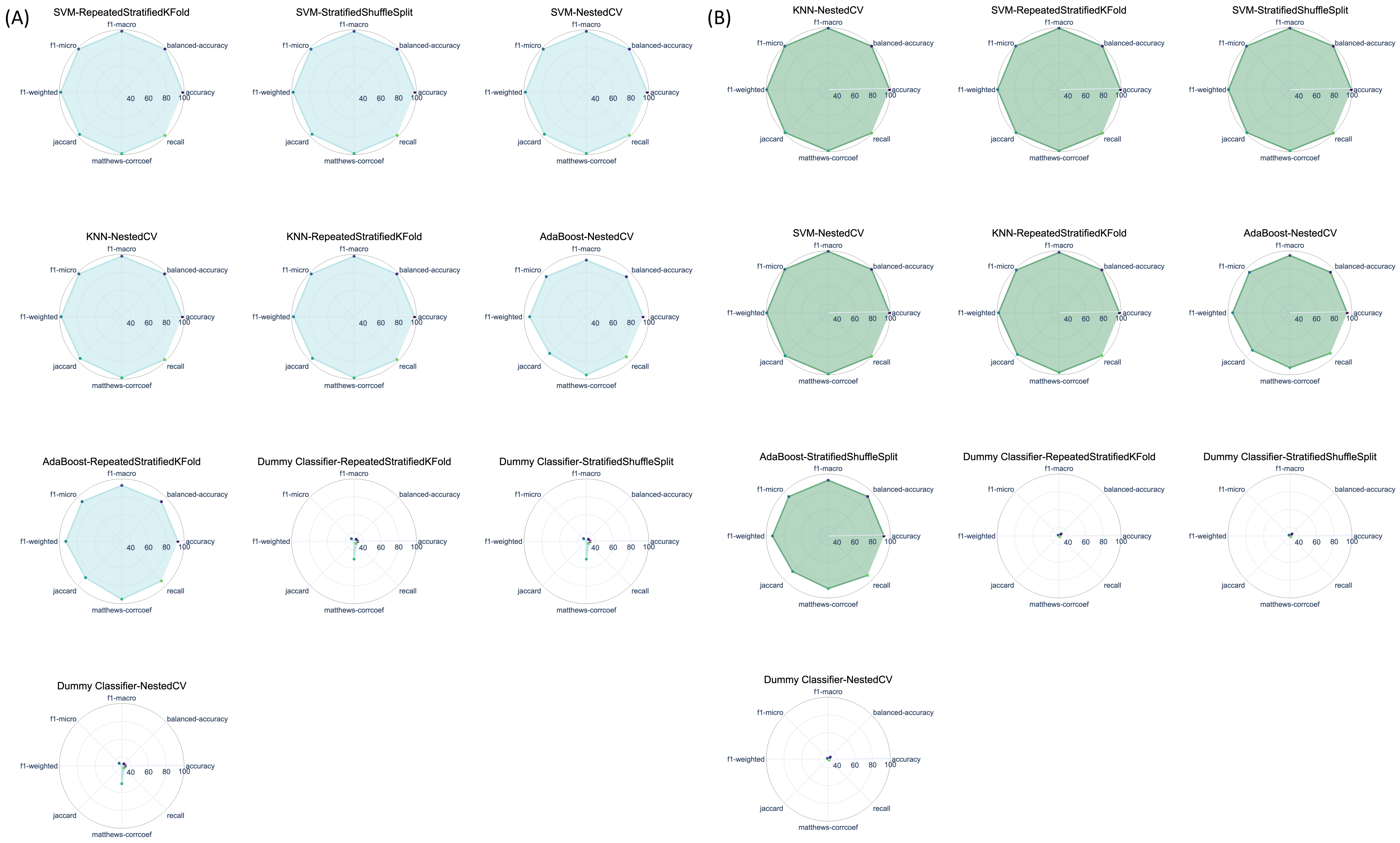

Figure S7. Projection of metrics scores on two-dimensional (2D) polar coordinates. The plots illustrate the performance scores of the top and worst five machine learning (ML) algorithms trained on the Peripheral Blood Mononuclear Cells (PBMC) dataset, both during training (A) and testing (B). Each ML model is represented by a circle, and each vertex represents a specific performance metric. A circle with a larger shaded area indicates better performance.

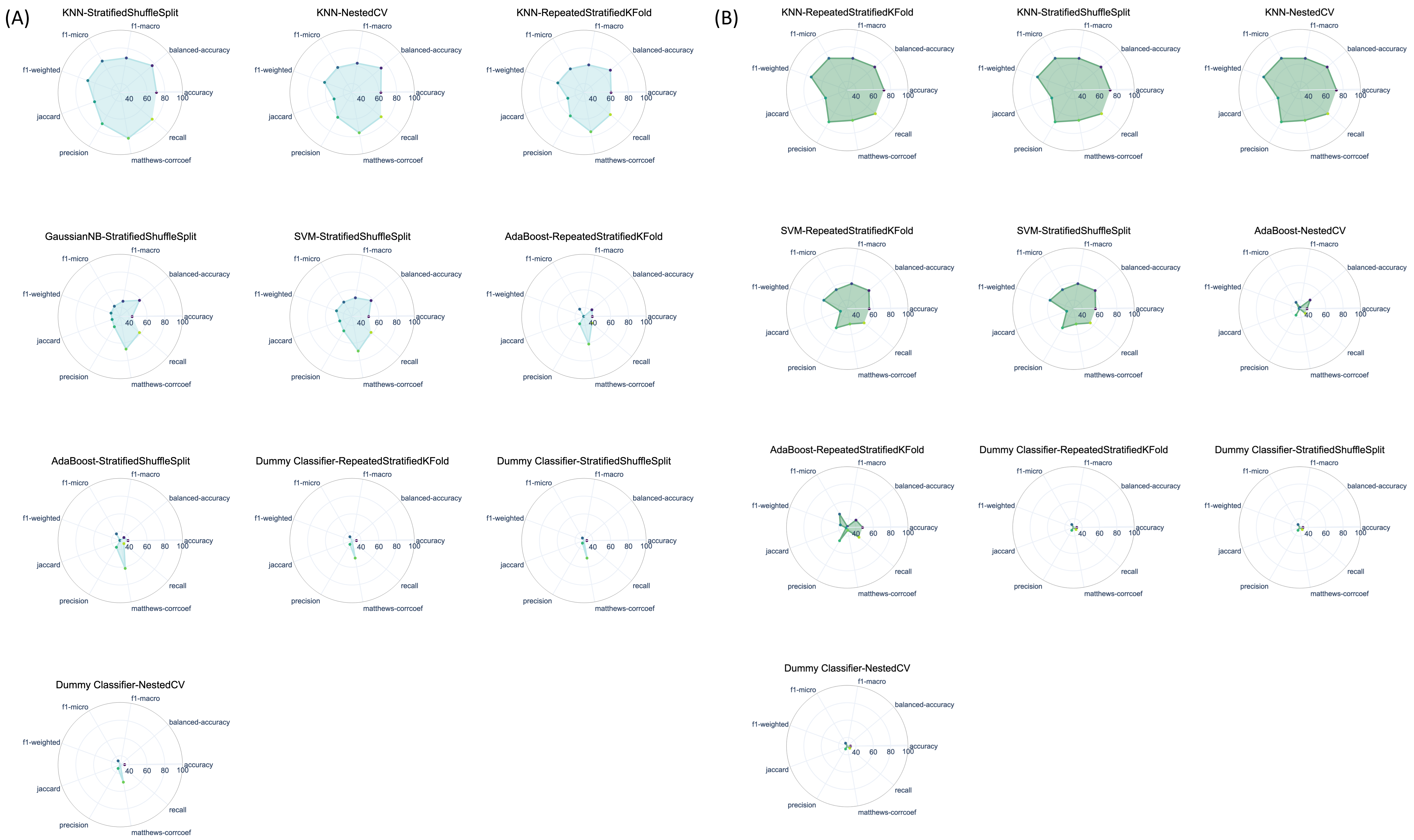

Figure S8. Projection of metrics scores on two-dimensional (2D) polar coordinates. The plots illustrate the performance scores of the top and worst five machine learning (ML) algorithms trained on the Glass Identification dataset, both during training (A) and testing (B). Each ML model is represented by a circle, and each vertex represents a specific performance metric. A circle with a larger shaded area indicates better performance.

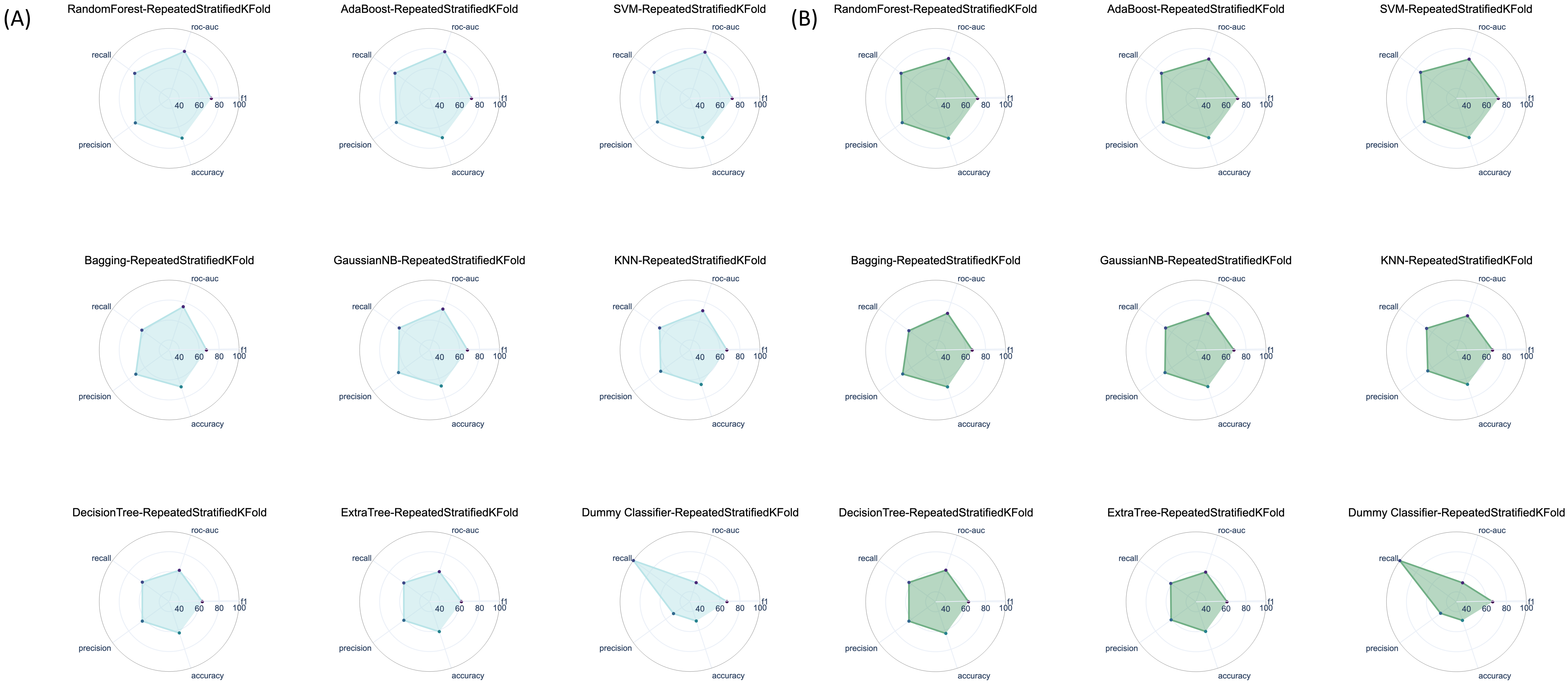

Figure S9. Projection of metrics scores on two-dimensional (2D) polar coordinates. The plots illustrate the performance scores of the machine learning (ML) algorithms trained on the Body Signal dataset, both during training (A) and testing (B). Each ML model is represented by a circle, and each vertex represents a specific performance metric. A circle with a larger shaded area indicates better performance.

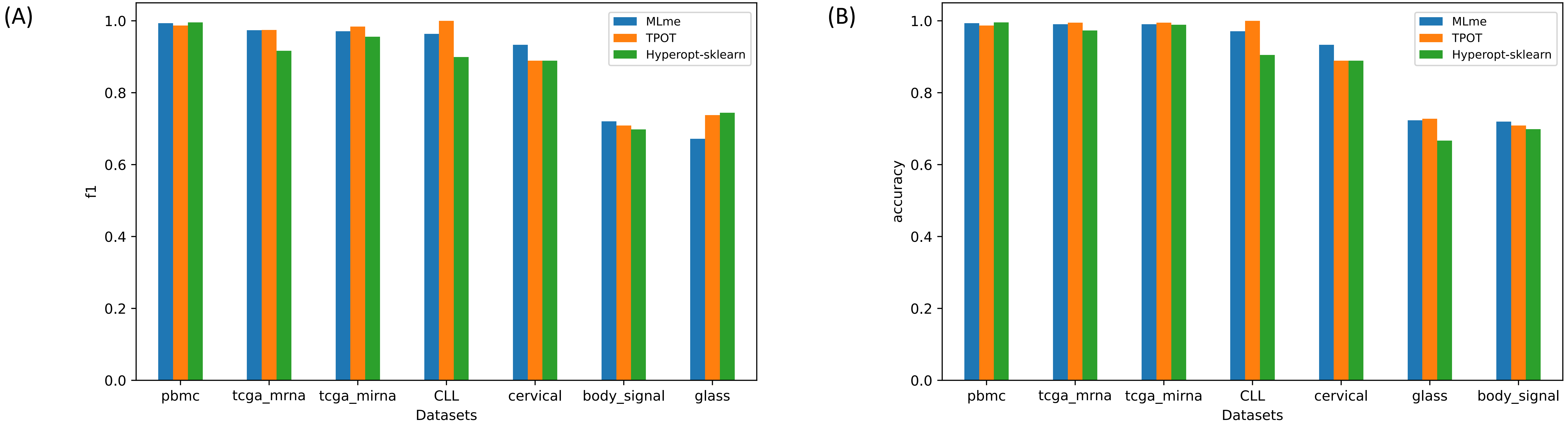

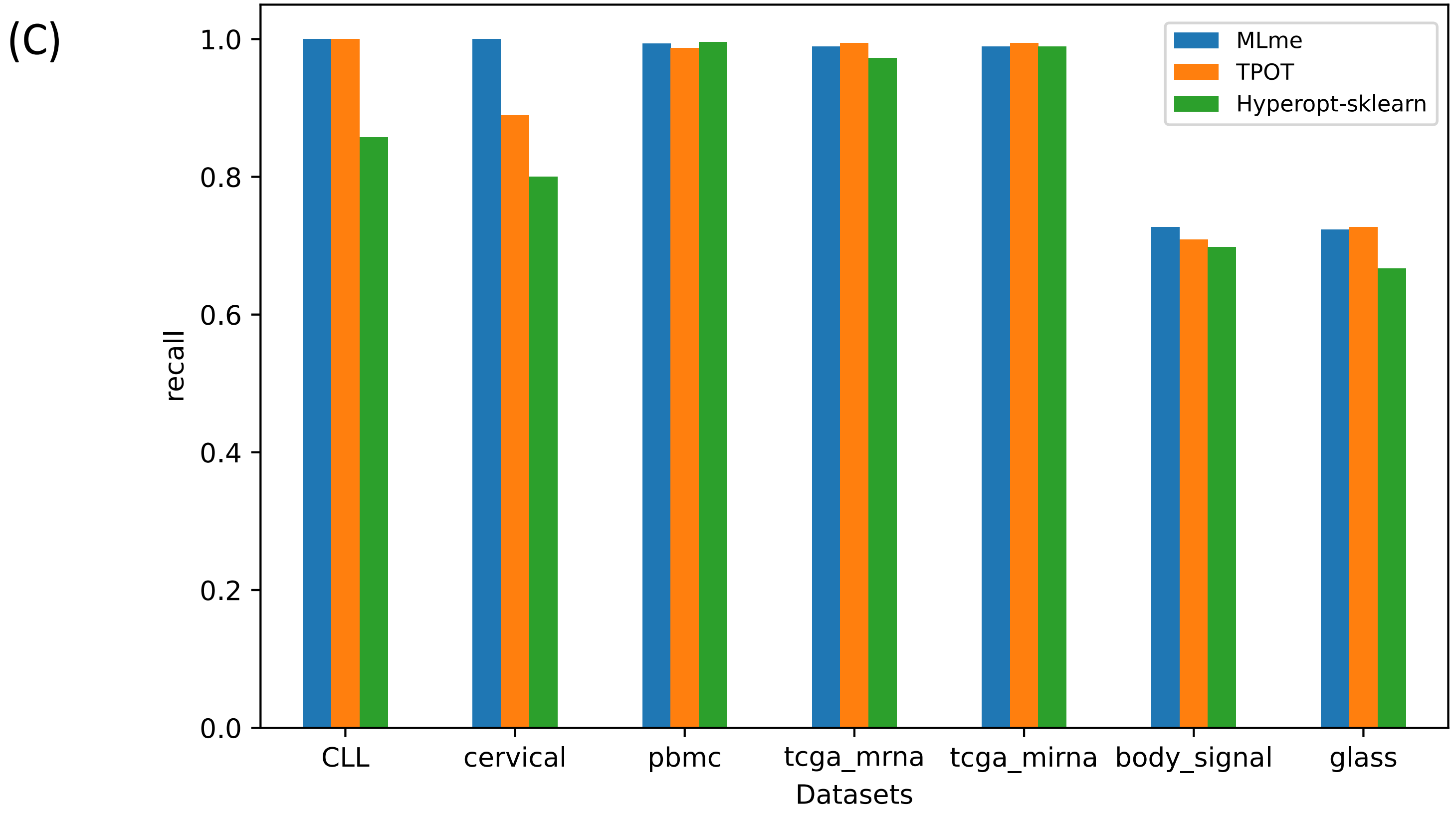

Figure S10. Performance Comparison of MLme, TPOT, and Hyperopt-sklearn across Multiple Datasets. Each bar in the figure (A), (B), and (C) represents the F1, accuracy, and recall scores respectively on the test data for each dataset.

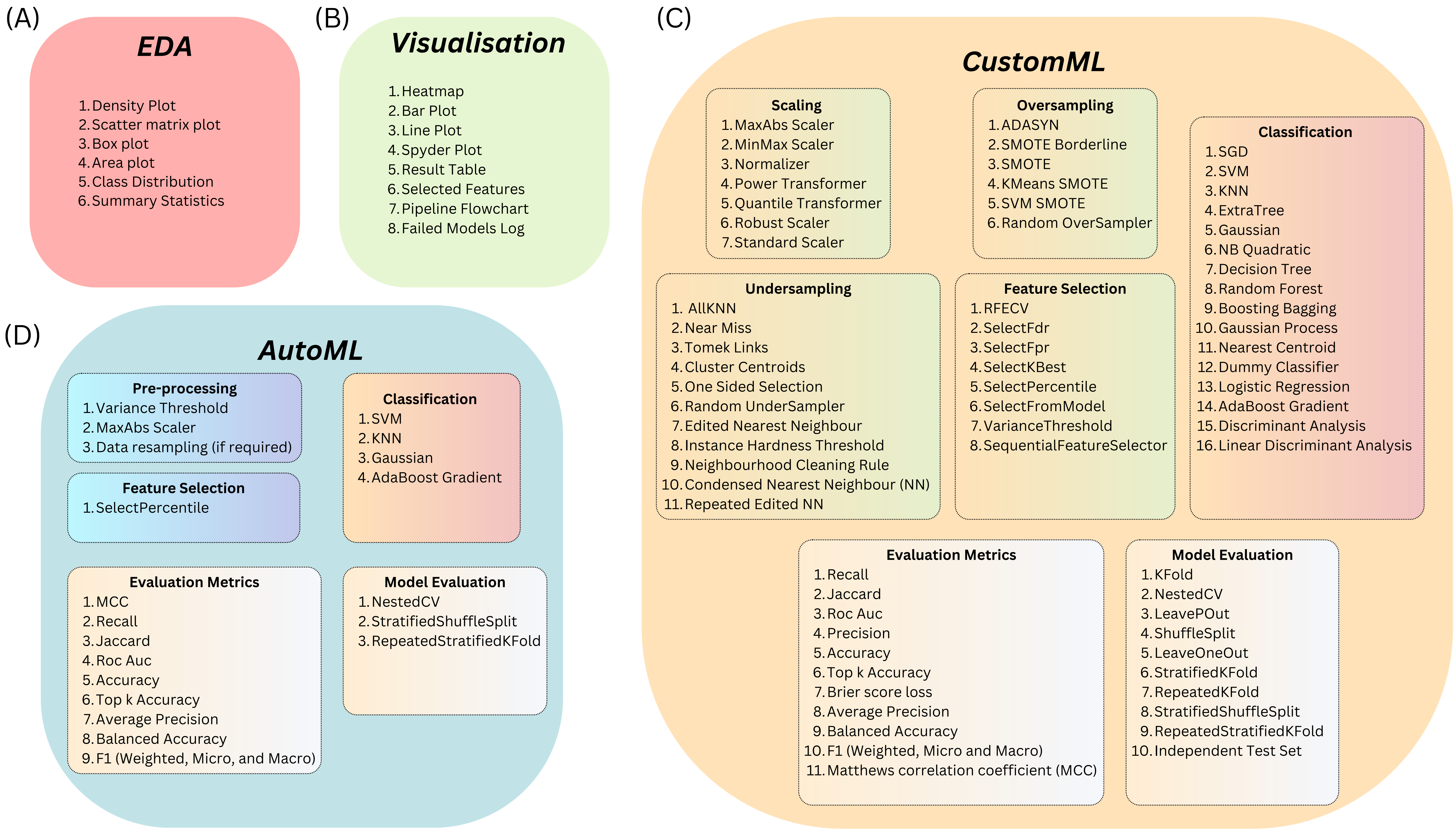

Figure S11: MLme's List of Algorithms and Diverse Plot Types for Different Machine Learning Stages. MLme offers an array of diverse plots suitable for both exploratory data analysis (EDA) **(A)** and visualizing outcomes derived from either autoML or customML (B). MLme provides users with the flexibility to design their own machine learning pipelines. Figure (C) illustrates the potential pipeline steps alongside corresponding algorithm choices. Furthermore, Figure (D) represents the steps and corresponding algorithms included in MLme's default autoML pipeline.
