# Supplementary material for: Machine Learning Made Easy (MLme): a comprehensive toolkit for machine learning–driven data analysis
Source: Gigascience. 2024 Jan 11;13:giad111. doi: 10.1093/gigascience/giad111 (PMC10783149; doi:10.1093/gigascience/giad111)
Supplement: giad111_Supplemental_Files [file giad111_supplemental_files.zip › supplementary_rev_4.docx]

Table S1: Comparison of features between MLme and other similar machine learning automation tools. * = Coding expertise is required. ^+^ = Only one algorithm can run at a time. N = Number of plots. GUI = Graphical User Interface.

| ***Dataset*** | ***Data type*** | ***Number of Samples*** | ***Number of Features*** | ***Target Class ratio*** |
| --- | --- | --- | --- | --- |
| CLL | mRNA | 136 | 5000 | Male (n=82): Female (n=54) |
| Cervical cancer | miRNA | 58 | 714 | Normal (n=29): Tumor (n=29) |
| TCGA-BRCA | miRNA | 1207 | 1404 | Normal (n=104): Tumor (n=1104) |
| TCGA-BRCA | mRNA | 1219 | 5520 | Normal (n=113): Tumor (n=1106) |
| PBMC | scRNA-seq | 1500 | 500 | CD8 Naive (n=500 cells) : CD14 Monocytes (n=500 cells) : CD16 Monocytes (n=500 cells) |
| Glass Identification | Oxide content (i.e., Na, Fe, K, etc) | 214 | 10 | Glass 1 (70), Glass 2 (76), Glass 3 (17), Glass 5 (12),  Glass 6 (10), Glass 7 (29) |
| Body signal | Body signal data (hemoglobin, triglyceride) | 100,000 | 21 | **Consume Alcohol**  Yes (n=﻿50173) : No (n= ﻿49827) |

*Table S2: Example datasets used in this study. CLL = Chronic Lymphocytic Leukemia. TCGA = The Cancer Genome Atlas. BRCA = Invasive Breast Carcinoma. PBMC = Peripheral Blood Mononuclear Cells.*

**Default ML Pipeline for AutoML**

The AutoML pipeline in MLme follows a sequence of operations (Figure 2). Below is a detailed breakdown of each step involved in the pipeline:

1. **Dataset Splitting:** The pipeline begins by splitting the input dataset into training and an independent test set (30 percent of the original dataset) if the user has activated the test set option. Otherwise, the entire dataset is used for training.
2. **Stratified Sampling:** The training data is then divided into n bins of equal size through stratified sampling. Among these bins, k-1 are assigned as training sets, while the remaining bin serves as the validation set.

All the steps, including feature selection and data resampling, will be performed solely on the training dataset after this split. The validation and test dataset (if available) will only be utilized for the model evaluation process.

1. **Pre-processing:**

- The first step is to remove the features with low variance. This involves eliminating any features with a variance lower than the threshold specified by the user via the user interface.
- Next, the MaxAbs and MinMax algorithms, utilizing default settings from Sklearn, are applied to maintain the distribution of the features within predefined ranges.
- To address data imbalances, the AutoML pipeline incorporates the RandomOverSampler and RandomUnderSampler algorithms from Sklearn, but only if selected by the user from the interface. These algorithms facilitate the generation of synthetic samples and the reduction of samples from the major class, respectively.

AutoML utilizes the GridSearchCV technique to determine the most appropriate scaling and resampling algorithm, from the algorithms mentioned, for a given dataset.

1. **Feature Selection:** The next step employs a univariate feature selection method called SelectPercentile to identify the essential features from the original set of features. The size of the resulting significant feature subset depends on the value specified by the user.
2. **Model Training:** AutoML trains five ML classification algorithms: Dummy Classifier, Support Vector Machine (SVM), K-Nearest Neighbors (KNN), AdaBoost, and Gaussian Naive Bayes (GaussianNB). All algorithms are used with default parameters from Sklearn.
3. **Model Evaluation:**

- An extensive array of evaluation metrics is incorporated, including accuracy, average precision, F1 score, balanced accuracy, macro and micro F1 scores, Jaccard score, MCC, recall, ROC-AUC score, and top K accuracy. These metrics collectively provide a comprehensive overview of the model's performance across various dimensions, ensuring a holistic evaluation process.
- The pipeline utilizes various evaluation techniques, including Repeated Stratified K-Fold (repeats=10, splits=5), Stratified Shuffle Split (splits=5), and Nested Cross-Validation (splits=5).

1. **Output Generation:** The pipeline produces a zip file containing the log .txt and results.pkl files as the final output. The results.pkl file contains the results of the AutoML pipeline, including the trained model, pipeline diagram, and evaluation scores. To ensure reproducibility, each step and algorithm will execute with a specific random seed provided.
2. **Result Examination:** Users can analyze the results by visualizing the contents of the pickle file using the visualization feature from MLme.
