# Supplementary material for: Machine Learning Made Easy (MLme): a comprehensive toolkit for machine learning–driven data analysis
Source: Gigascience. 2024 Jan 11;13:giad111. doi: 10.1093/gigascience/giad111 (PMC10783149; doi:10.1093/gigascience/giad111)
Supplement: giad111_Response_to_Reviewer_Comments_Original_Submission [file giad111_response_to_reviewer_comments_original_submission.pdf]

Author's Response To Reviewer Comments

Dear Editors,

I would like to express our sincere gratitude for the valuable comments provided by the reviewers of our manuscript submitted to Gigascience Journal. Their constructive feedback has been instrumental in enhancing the quality of our work, and we are grateful for their time and insights.

We have carefully addressed the reviewers' comments and made significant revisions to the manuscript to incorporate their suggestions. Specifically, we have registered our software application in the bio.tools and SciCrunch.org databases, as requested, and have included the corresponding RRID and biotoolsID identifiers in the manuscript, as outlined in the reviewers' feedback.

We have also responded comprehensively to each of the reviewers' major and minor concerns, providing detailed explanations and making the necessary adjustments to our manuscript and software where applicable. We have included additional comparisons with other tools, expanded the feature descriptions, and improved transparency regarding our pipeline's functionalities.

Furthermore, we have taken the reviewer's suggestions to heart and have removed the default data resampling option, providing users with the ability to choose whether to include it in their workflow. We believe this adjustment enhances transparency and user control.

Additionally, we have included a larger dataset to showcase MLme's versatility and applicability across various data sizes and types.

Once again, we would like to express our appreciation for the reviewers' efforts in evaluating our work. We believe that the revisions we have made address their concerns and strengthen our manuscript. We are confident that these improvements will enhance the value of our contribution to the field of machine learning-driven analysis.

Thank you for your consideration.
Ali Hashemi

--------------------------------------- Editor's Comment
In addition, please register any new software application in the bio.tools and SciCrunch.org databases to receive RRID (Research Resource Identification Initiative ID) and biotoolsID identifiers, and include these in your manuscript. Computational workflows should be registered in workflowhub.eu and the DOIs cited in the relevant places in the manuscript. These will facilitate tracking, reproducibility and re-use of your tool.

........................Response to Editor's Comment ....
Thank you for your valuable feedback. In compliance with your request, we have included these unique identifiers in the manuscript. They can be found at line 401 and 402 in main manuscript under Availability of supporting source code and requirements.

BioTool ID: MLme
https://bio.tools/mlme

SciCrunch ID: MLme (RRID: SCR_024439)
https://scicrunch.org/resources/about/registry/SCR_024439

Workflow: https://workflowhub.eu/workflows/571?version=1
(cited in line number 189)

###
Reviewer 1
###

-- Reviewer's Comment
Reviewer #1: Akshay et al. present MLme, a toolkit for exploring data and automatically running machine learning (ML) models. This software could be useful for those with less experience in ML. I believe it is suitable for publication provided the following points are addressed.

.........................Response to Reviewer's Comment ....
Thank you for your time and thoughtful feedback on our manuscript about MLme. Your contribution has been instrumental in improving the quality of our manuscript, and we greatly appreciate your insights. Below, you will find our responses to each of your comments.

Major

-- Reviewer's Comment

1. The performance of models is consistently over 90% but without a reference point it is unclear how good this is. Are there results from previous studies on the same data that can be compared to, with a table comparing accuracy with MLme to previous work? Otherwise it is unclear whether MLme is supposed to be a quick way to have a first go at prediction on the data or can entirely replace manual model refinement.

.........................Response to Reviewer's Comment ....
Thank you for your valuable input. We appreciate your suggestion and have taken it into careful consideration. We have conducted a comparison between MLme, TPOT, and hyperopt-sklearn, and as expected, the results from both tools demonstrate comparability. For specific details and results, please refer to lines 307 to 315 and Figure S9.C . We believe this additional information will provide valuable insights to our readers.

--Reviewer's Comment
2. With any automated ML system it is important to impress upon users the risks of ML. For example, the splitting of data into training and test sets is done randomly, but there are cases where this is not appropriate as it will lead to data leakage between the training and test sets. This could be mentioned in the manuscript and somewhere on the GUI. There isn't really a replacement for domain knowledge, and users of MLme should have this in mind when using the software.
.........................Response to Reviewer's Comment ....
Thank you for your feedback and valuable suggestion regarding the inclusion of information on the risks associated with the automated ML tools. We have taken your input seriously, and we are pleased to inform you that we have incorporated the corresponding text into the Conclusion section of our manuscript, spanning from line 371 to 376.

Minor
--Reviewer's Comment
3. More experienced ML users may want to use the software to have a first go at prediction on the data. For these users it may be useful to provide access to commands or scripts, or at least information on which functions were used, as additional options in the GUI. Users could then run these scripts themselves to tweak hyperparameters etc.

.........................Response to Reviewer's Comment ....
Certainly, we appreciate your interest in enhancing the utility of MLme! We are pleased to inform you that we have already made the autoML script publicly available on the MLme GitHub repository (https://github.com/FunctionalUrology/MLme/blob/main/subScript.py). Additionally, CustomML also provides the scripts, a readme file, and allows users to select values for each hyperparameter of all the

available algorithms.

--------------------------------------Reviewer's Comment
4. The visualisation tab lacks an info button by the file upload to say what the file format should be.
........................Response to Reviewer's Comment ....
Thank you for bringing this to our attention. We have made an update to the visualization tab. An "info" button has been added next to the file upload section to provide clear guidance on the acceptable file formats.

##
Reviewer 2
##

--------------------------------------Reviewer's Comment
Reviewer #2: In this paper the authors introduce MLme, a comprehensive toolkit for machine-learning driven analysis. The authors discuss the benefits and limitations of their toolkit and provide a demonstration evaluation on 6 datasets suggesting it's potential value. Overall MLme seems like a nice, easy to use tool with a good deal of potential and value.

........................Response to Reviewer's Comment ....
Thank you for your time and thoughtful feedback on our manuscript about MLme. Your contribution has been instrumental in improving the quality of our manuscript, and we greatly appreciate your insights. Below, you will find our responses to each of your comments.

--------------------------------------Reviewer's Comment
- However, as the developer of STREAMLINE, an AutoML toolkit with a number of very similar goals and design architecture to MLme it was very surprising to have it not referenced or compared to in this paper. My major concerns involve the limited details about what this specifically does/includes (e.g. what 16 ML algorithms are built in), as well as what seems like a limited and largely biased comparison of this toolkit's capabilities to other autoML tools (most specifically STREAMLINE which has a significant degree of similarity).

........................Response to Reviewer's Comment ....
We appreciate the reviewer for bringing STREAMLINE to our attention and for taking the time to provide valuable and constructive feedback.
Indeed, STREAMLINE and MLme share common goals and address similar problems in the field of ML. We acknowledge that STREAMLINE offers some important features, such as data imputation and automatic hyperparameter tuning, which are currently not available in MLme. We appreciate the opportunity to learn about these features and will consider them for potential future enhancements to our toolkit.
One of the distinctive features of MLme is its emphasis on providing users with a code-free experience while offering extensive control over the design of machine learning pipelines. This feature sets MLme apart from other AutoML tools, including STREAMLINE. Additionally, we would like to emphasize that MLme is not solely an AutoML tool; it is a comprehensive platform encompassing data exploration, AutoML, custom ML pipeline design, and result visualization. All these functionalities are designed to be user-friendly and interactive, facilitated by an intuitive user interface.
With these features, MLme aims to cater to users with limited programming experience, providing accessibility to machine learning for those who may not possess advanced coding skills. Even for experienced users, there is a steep learning curve to use the other available tools. While STREAMLINE also strives to achieve this goal to some extent, it still requires users to have coding expertise.
Initially, we did not include tools in our comparison table (S1) that demand programming expertise, with

the exceptions of Hyperopt and Autosklearn, due to their popularity in the field. However, in response to your comment, we have endeavored to provide a fair and comprehensive comparison between MLme and the other tools, as detailed in table S1.
For a more detailed understanding of the algorithms and steps integrated into MLme, please refer to figure S10 (line 235-237).

--------------------------------------Reviewer's Comment
- There are many other autoML tools out there that that authors have not considered in their Table S1 or referenced. Eg. MLBox, AutoWeka, H20, Devol, Auto-Keras, TransmorgriffAI, and most glaringly in for this reviewer, STREAMLINE (https://github.com/UrbsLab/STREAMLINE).

.........................Response to Reviewer's Comment ....
We value the feedback from the reviewers and believe that the expanded Table S1 has effectively addressed your concerns.
We would like to mention that, Devol is not an autoML tool but rather a tool for automatically building neural networks. Therefore, it cannot be compared to MLme or other tools mentioned here.

--------------------------------------Reviewer's Comment
- In particular, with respect to STREAMLINE (https://link.springer.com/chapter/10.1007/978-981-19-8460-0_9), there are a large number of pipeline similarities and a similar analysis mission/goals to MLme that make it extremely relevant to cite and contrast to in this manuscript as well as in Table S1. STREAMLINE has a similar focus on the end-to-end ML analysis pipeline including automated exploratory analysis, data processing, feature selection, ML modeling with 16 algorithms, evaluation, and results visualization generation, interactive visualizations, pickled output storage, etc. The first STREAMLINE paper was published March of 2023, and a preprint of that manuscript published June 2022, as well as a precursor implementation of this pipeline published as a preprint in Aug of 2020 (https://arxiv.org/abs/2008.12829). This in contrast with MLme's preprint published July of 2023. While MLme has a number of potentially nice features that STREAMLINE does not (i.e. a GUI interface, spider plots, easy color palate selection, inclusion of a dummy classifier, ability to handle multi-class classification [which is not yet available, but in development for STREAMLINE along with regression]), it lacks other potentially important features that STREAMLINE does have (i.e. automated hyperparameter optimization, basic data cleaning and feature engineering [in the newest release], collective feature selection, pickled models for later reuse, collective feature importance visualizations, a pdf analysis summary report, the ability to quickly evaluate models on new replication data, and potentially other capabilities that I can't highlight because of limited details on what MLme includes). The absence of hyperparameter optimization is a particularly problematic omission from MLme, as this a fairly critical element of any machine learning analysis pipeline.

.........................Response to Reviewer's Comment ....
We sincerely appreciate your thorough review and valuable insights.
Regarding the absence of automated feature engineering in MLme, we firmly believe in the importance of domain expertise in this process. Different datasets and domains often require tailored feature processing approaches. For instance, in proteomics datasets, where missing values are prevalent, a simplistic mean/median imputation might not suffice. We believe that users should have control over these crucial decisions to ensure the best outcomes. This approach empowers users to make informed choices based on their specific data characteristics and domain knowledge.
Regarding hyperparameter optimization, we acknowledge its significance in machine learning pipelines. This feature is slated for future updates, as outlined in our manuscript (lines 385 to 391). In the current version of MLme, users can choose values for every parameter across all the available algorithms.
Furthermore, MLme offers a range of functionalities, including feature selection, pickled models for future use and evaluation on new data, and interactive result visualizations. Our visualizations are designed to be dynamic and publication-ready, providing users with a more interactive and informative experience compared to static reports.
Once again, we appreciate your feedback, which will undoubtedly help us enhance MLme's capabilities and usability for the research community.

--------------------------------------Reviewer's Comment
-Table S1 should be expanded to highlight a broader range of toolkit features to better highlight the strengths and weaknesses of a greater variety of methodologies. The present table seems a bit cherry picked to make MLme stand out as appearing to have more capabilities than other tools, but there are uncaptured advantages to these other approaches.

........................Response to Reviewer's Comment ....
Thank you for your feedback. We have attempted to address this by expanding Table S1.

--------------------------------------Reviewer's Comment
-This manuscript includes no citations justifying their pipeline design choices. In particular, I'm most concerned with the author's justification of automatically including data resampling by default as it is well known that this can introduce bias in modeling. It's also not clear what determines if data resampling is required, and whether this only impacts training data or also testing data. Its not clear that resampling is a good/reliable strategy for an automated machine learning framework since data resampling to create a more balanced dataset can also incorporate bias in to an ML model.

........................Response to Reviewer's Comment ....
Thank you for your thoughtful feedback, and we appreciate your comments regarding the inclusion of data resampling by default.
Initially, our intention was to simplify the user experience in our autoML feature by limiting the number of available options. We recognized that the abundance of choices could be overwhelming for newcomers. Therefore, we implemented data resampling internally, but only when the class imbalance was not hugely skewed. Our approach leveraged the pipeline functionality from scikit-learn to ensure that there was no data leakage, meaning that the test dataset remained independent of all preprocessing steps throughout the process. Additionally, autoML provided users with multiple evaluation metrics, including those capable of detecting bias introduced by resampling, such as Precision, Recall, and ROC AUC. We assumed that users would have the expertise to interpret these metrics.
However, your feedback has prompted us to reconsider the potential biases introduced by resampling, especially when users may not have the proficiency to evaluate its effects. In response to your suggestion, we have decided to remove the default resampling option altogether. Instead, we will provide users with a toggle button, allowing them to choose whether they want to include oversampling in their workflow.
We believe that this adjustment will enhance the transparency and control that users have over the training process while mitigating the risk of unintended bias. Your input has been invaluable in improving our platform, and we thank you for your constructive feedback.

--------------------------------------Reviewer's Comment
- In the context of potential datasets from different domains (including biomedical data), the datasets identified in this paper as being "large" have only up to 1500 sample and only up to 5520 features, which would not be considered large by most data scientist standards.

........................Response to Reviewer's Comment ....
Thank you for your feedback. Indeed, a sample size of 1500 may not be considered large in today's data landscape. However, it's worth noting that in the realm of biological human samples, this dataset is one of the largest and relatively rare. Additionally, machine learning algorithms tend to perform well on larger datasets, but they can encounter challenges when dealing with smaller sample sizes or data suffering from the curse of dimensionality. Hence, we intentionally focused on these types of datasets to thoroughly test MLme's capabilities in such scenarios.
To address your concern and showcase the versatility of MLme, we have included a larger dataset comprising biometric data from 100,000 individuals, collected from the National Health Insurance Service in Korea (line 291-295). Our aim was to predict smoking status based on this extensive biometric dataset.

For more detailed information about this dataset, please refer to Table S2. You can also find the corresponding in Figure S9 (A and B). We hope this addition provides a more comprehensive view of MLme's applicability across various dataset sizes and types.

---------------------------------------Reviewer's Comment
- There are largely limited details in this paper and the software's github documentation in terms of transparently indicating exactly what this pipeline does, and what options, algorithms, evaluation metrics, and visualizations it includes.

........................Response to Reviewer's Comment ....
We appreciate your valuable feedback regarding the transparency of MLme's default pipeline and the available documentation.
We have already included the details about the default pipeline (Figure 2) used by the autoML. Furthermore, in response to your suggestion, we have taken action to enhance the clarity of MLme's functionality. Specifically, we have added an additional figure (Figure S.10, Line 235 to 237) that elucidates the list of available algorithms/steps and diverse plot types for different machine learning stages. This addition aims to provide a more comprehensive overview of the options, algorithms, evaluation metrics, and visualizations integrated into our software.

---------------------------------------Reviewer's Comment
- Since the authors do not benchmark MLme against any other autoML tool and they have a very limited set of benchmarked datasets (6 total, with limited diversity of data types, sizes, feature types), I don't think it's fair to claim that their methodology necessarily excels in it's core objective of addressing classification tasks. Ideally the authors would conduct benchmarking comparisons to STREAMLINE, as well as other autoML toolkits, however this might also understandably be outside the scope of this current paper. I do suggest the authors be more conservative in what assertions they make and conclusions they draw with respect to MLme. The authors might consider using established ML or AutoML benchmark benchmark datasets used by other algorithms and frameworks to compare or facilitate comparison of their pipeline toolkit to others.

........................Response to Reviewer's Comment ....
Thank you for your valuable input. Here, it's important to clarify that MLme doesn't introduce a new methodology; rather, it leverages established ML algorithms like SVM and KNN at its core. As a result, we anticipate similar performance to other automatic ML tools.
To address your concern, we have conducted a comparison between MLme, TPOT and hyperopt-sklearn, and as expected, the results from both tools demonstrate comparability. For specific details and results, please refer to lines 307-315 and Figure S9.C . We believe this additional information will provide valuable insights to our readers.
