# Supplementary material for: Machine Learning Made Easy (MLme): a comprehensive toolkit for machine learning–driven data analysis
Source: Gigascience. 2024 Jan 11;13:giad111. doi: 10.1093/gigascience/giad111 (PMC10783149; doi:10.1093/gigascience/giad111)
Supplement: giad111_Response_to_Reviewer_Comments_Revision_1 [file giad111_response_to_reviewer_comments_revision_1.pdf]

Author's Response To Reviewer Comments

###################################
######## Reviewer comment #########
###################################
Reviewer #2: Overall I think the authors have made some good improvements to this paper, although it does not seem like the main body of the paper has changed much with most of the updates going into supplemental materials. However, I think this work is worthy of publication once the following items are addressed. (which I still feel strongly should be addressed, but should be fairly easy to do so).

--------------- Our Response -----------------

We appreciate reviewer's feedback. We have gone through the reviewer's feedback and changed the manuscript and supplementary accordingly.

###################################
######## Reviewer comment #########
###################################
1. Limitations section: While the authors added some basic comparisons to a few other AutoML tools, I do not see how they are justified in saying that MLme 'excells' in it's core objective of addressing classification tasks. This implies it is better performing a classification than other methods, which is not at all backed up here, and indeed would be very difficult to prove as it would require a huge amount of analyes over a broad range of simulated and real world benchmark datasets, and incomparison to many or all orther other autoML tools. At best i think the authors can say here that it is at least comparable in performance to AutoML tools (X, Y, Z) in its ability to conduct classification analyses. And according to Figure S9 this is only across 7 datasets, and focused only on the F1 score which could also be missleading or cherry picked. At best I believe the authors can say in the paper that "Initial evaluation across 7 datasets suggested that MLMe performed comparably to TPOT and Hyperopt-sklearn with respect to F1 score performance. This suggests that MLme is effective as an automated ML tool for classification tasks. " (or something similar).

--------------- Our Response -----------------

The first line highlighted by the reviewer, 'MLme excels in its core objective of addressing classification tasks,' is from the limitations section (line number 350-352), where we mentioned that MLme lacks functionality for regression and clustering problems. However, it is well-equipped to handle 'pipeline development' for classification tasks. We have updated this text to avoid any further confusion.
The second part of the reviewer's comment related to the results section where we compared MLme with other tools. We would like to reiterate that we never claimed that MLme performs better in terms of performance, but rather emphasized its superiority in terms of ease of use such as visualization and exploratory analysis.
Regarding the last part about updating the text, we would like to clarify that we have already used the word 'similar' in our text (line number 309) when discussing the performance comparison and we have been avoiding to claim as 'better'. Furthermore, we would like to emphasize that we did not cherry-pick the F1 metric; instead, it is one of the commonly (recommended) used metrics to use for the nature of the datasets (imbalanced) we had. However, to address the reviewer's concern, we have included accuracy and recall scores as well. MLme demonstrates similar performance based on these metrics as well (Figure S10).

###################################
######## Reviewer comment #########
###################################
2. While the authors lengthened the supplemental materials table comparing ML algorithms (mainly by

adding some other autoML tools, this table is intentionally presenting the capabilities of tools in a way that make it appear like MLme does the most (with the exception of the 'features' column) . For example, what about a column to indicate if an autoML tool has an automated pipeline discovery component (like TPOT)? In terms of AutoML, this table is structured to highlight the benefits of MLme, rather than give a fair comparison of AutoML tools (which is my major concern here). In terms of AutoML performance and usability there is alot more to these different tools than the 6 columns presented. In this table 'features' seems like an afterthought, but is arguably the most important aspect of an AutoML.

--------------- Our Response -----------------

We want to reemphasize that MLme is not just an autoML tool; rather, autoML is one of the features offered by MLme. Regarding the reviewer's comment about TPOT having an automated pipeline discovery component, we assume the reviewer meant that TPOT can run multiple algorithms with different parameters and provide the best performing algorithm/parameters for your datasets. We have already included this information in the introduction (line 137-139), where we mentioned that most autoML tools function as algorithm recommenders, whereas MLme does not provide this functionality.

###################################
######## Reviewer comment #########
###################################
3. Additionally, the information presented in the autoML comparison table does not seem to be entirely accurate, or at least how the columns are defined is not made entirely clear. Looking at STREAMLINE, which can be run by users with no coding experience (as a google colab notebook), it has a code free option (just not a GUI), STREAMLINE also generates more than two exploratory analysis plots, and more results visualizations plots than indicated). While I agree that MLme has many more ease of use functionality in comparison to STREAMLINE (which is a very nice plus), a reader might look at this table and think they need to know how to code in order to use STREAMLINE, which is not the case. Could the authors at least define their criteria for the "code free" column. As it's presented now it seems to be the same exact criteria as for GUI (in which case this is redundant). The same is true for the legend for the table where '*' indicates that coding experience is required for designing a custom pipeline. This requires more clarification, as STREAMLINE can be customized easily without coding experience by simply changing options in the Google Colab notebook, and TPOT automatically discovers new analysis pipelines which isn't reflected at all.

--------------- Our Response -----------------

We have run the STREAMLINE and tried to compare the functionality and similarities with other tools but unfortunately, we were not able to acquire the information about the number of plots generated by STREAMLINE in the paper or on GitHub. Therefore, we used the number of plots generated by Google Colab as the total number of available plots in STREAMLINE. However, to avoid any confusion, we have removed this information from the table for all the tools.
Regarding the second point about coding expertise, we truly appreciate this comment from the reviewers. However, we believe whether coding expertise is required or not in these scenarios is a subjective question. In the context of STREAMLINE, we believe that having a basic familiarity with Python coding can enhance its usage, although it's worth noting that the primary focus is on adjusting variable values. These variables are tied to Python code and should be modified within a Python notebook. While this process might be manageable for machine learning experts and bioinformaticians, it could pose a challenge for users less experienced with coding. Particularly, when faced with a notebook containing numerous lines of code and the need to modify multiple parameters based on specific datasets or problems, it might seem a bit overwhelming. As previously mentioned, the perception of how code-free a solution is ultimately depends on individual viewpoints, which can be better assessed through user surveys. Therefore to address the reviewers' concern about redundancy in the columns, we have removed the 'code-free' column for now.
###################################
######## Reviewer comment #########
###################################

4. While I appreciate the authors adding a citation for STREAMLINE and some other autoML tools not previously cited, it would be nice for the authors to discuss other AutoML tools further in their main paper, as well as to acknowledge in the main paper which AutoML tools are most similar to MLme in overall design and capabilities. Based on my own review of AutoML tools the most similar tools would include STREAMLINE and MLIJAR-supervised.

--------------- Our Response -----------------

Firstly, we'd like to express our gratitude for the reviewer's valuable comment. We'd like to emphasize that since this manuscript is designed as an application note, it naturally leans towards brevity. We believe that providing an extensive comparison of these tools in the main text might go beyond the current scope of this manuscript. However, there's potential to explore a more in-depth comparison in a future review paper, which could be an exciting avenue to consider.
####################################
######## Reviewer comment #########
####################################
5. I like the addition of Figure S10 that more clearly lays out the elements included in MLme, but I still think the paper and documentation lacks a clear and transparent walk through of exactly what happens to the data and how the analyses are conducted from start to finish when using the AutoML (at least by default). This is important to trusting what happens under the hood for reporting results, etc.

--------------- Our Response -----------------

The pipeline design for the autoML has already been provided (please refer to Figure 2).

####################################
######## Reviewer comment #########
####################################

Other comments responding to author responses:
* I still disagree with the authors that a dataset with up to 1500 samples or up to 5520 features could be considered large by today's standards across all research domains. Even within biomedical data, datasets up to 100K subjects are becoming common, and 'omics' datasets regularly reach hundreds of thousands to multiple millions of features. I am glad to see the authors adding a larger dataset, but i would still be cautions when making suggestions about how well MLme handles 'large' datasets without including specifics for context. However ultimately this is subjective, and not preventing me from endorsing publication.

--------------- Our Response -----------------

We appreciate the reviewer's understanding that this is a subjective matter, and we hope that our paper, with the inclusion of a larger dataset and the context provided, will offer valuable insights to a wide audience of researchers.
####################################
######## Reviewer comment #########
####################################
* I also disagree that MLme isn't introducing a new methodology. The steps comprising an AutoML tool can be considered in itself a new methodology, even if it is built on established components, because there are still innumerable ways to put a machine learning analysis pipeline together that adds bias, data leakage, or just yields poorer performance. Thus I also don't think it's fair to just 'assume' your method will work as well as other AutoML tools, especially when you've ran it on a limited number of datasets/problems.

--------------- Our Response -----------------

We appreciate the reviewer's comment. While we intended to emphasize that the core components of

MLme draw upon established techniques, we recognize that the unique combination and configuration of these components within our framework can indeed be considered a methodology in its own right.
