# Supplementary material for: Machine Learning Made Easy (MLme): a comprehensive toolkit for machine learning–driven data analysis
Source: Gigascience. 2024 Jan 11;13:giad111. doi: 10.1093/gigascience/giad111 (PMC10783149; doi:10.1093/gigascience/giad111)
Supplement: giad111_Response_to_Reviewer_Comments_Revision_2 [file giad111_response_to_reviewer_comments_revision_2.pdf]

# Author's Response To Reviewer Comments

###################################
######## Editor comment #########
###################################
Overall, the reviewer and I agree the tool has merit and the paper could be ultimately suitable for publication. However, the reviewer insists that the manuscript should not contain “any claims they are making that their methodology is superior (in any way) to other AutoML tools without sufficient evidence to support this claim", and I agree that the wording in the paper still needs adjustment to respond to this important concern.
-----------------------------------------------------
--------------- Our Response -----------------
-----------------------------------------------------
Thank you for handling our manuscript and highlighting the concerns expressed by the reviewer. In light of the reviewer's critical comment, we have made the necessary adjustments to the manuscript's wording, as recommended. The phrase "excels in its primary objective" has been updated to "well-equipped to develop pipelines for classification tasks", as recommended by reviewer. Please refer to line number 350-351. We hope that this adequately addresses the reviewer's concerns.

###################################
######## Reviewer comment #########
###################################
Critical Issue:
Limitations section: The authors updated the text - "excels in it's core objective of addressing classification tasks." To "it excels in its primary objective of addressing pipeline development for classification tasks. The use of the word 'excels' is the key problem, as this word is defined as "to do or be better than others". While the change in phrasing correctly no longer implies that MLme performed better than the other evaluated AutoML tools, it does still imply that it is the best in developing a pipeline for classification tasks, but no specific evidence is provided in the paper to support this assertion. I.e. there were no studies comparing how easy the tool was for users to apply than other autoML, and no detailed comparison of what pipeline elements could be included by MLme vs other autoML or pipeline development tools. The fact that MLme doesn't include hyperparameter optimization is in itself a limitation that I think would prevent MLme from being claimed as excelling or superior in pipeline development to other tools/platforms, even if it's easier to use that other tools. As phrased in the reviewer response, the authors could say that MLme is well-equipped to handle pipeline development as this would be a fair statement. All together I'd strongly encourage the authors not to make statements about the superior aspects of MLme without clearly backing up these statements with direct comparisons. Instead I'd suggest highlighting elements of MLme that are 'unique' or provide more functionality in contrast with other tools. In the reviewer response the authors make the claim that MLme is superior in terms of ease of use for visualization and exploratory analysis. If they want to make that statement in the paper backed up by accurate comparisons to other tools, I'd agree with that addition.
-----------------------------------------------------
--------------- Our Response -----------------
-----------------------------------------------------

Thank you for your valuable and insightful feedback regarding the language used in our limitations section. We have taken reviewer's suggestion and made the necessary adjustments. The phrase "excels in its primary objective" has been updated to "is well-equipped to develop pipelines for classification tasks." Please refer to line number 350-351. We hope that this adequately addresses the reviewer's concerns.

###################################
######## Reviewer comment #########
###################################
Non-Critical Issues that I feel still should be addressed:

1. Table S1 has been updated to remove the inaccuracies I previously pointed out, however this alone does not change the broader concern I had regarding the intention of this table (which is to highlight the parts of MLme that appear better than other AutoML tools without fairly pointing out the limitations of MLme in contrast with other tools). As a supplemental materials table, I do not feel this is critical, but I think making a table that more fairly reflects strengths and limitations of different tools would greatly strengthen this paper.

-----------------------------------------------------

--------------- Our Response -----------------

-----------------------------------------------------

We have already included the essential features provided by other tools, which are not available in MLme. However, we are not entirely sure if the reviewer's concern related to the content or the layout of the table. We presume that it is related to the table's layout, and therefore, we have made adjustments to ensure that the information is presented clearly and in unbiased manner. Please refer to table S1 in supplementary file. We hope that this adequately addresses the reviewer's concerns.

###################################

######## Reviewer comment #########

###################################

2. The pipeline design in Figure 2 and S10 are both high-level and still do not provide enough detail/clarity to understand exactly what happens and in what order when applying the autoML element of MLme. They key words here being transparency and reproducibility. The supplemental materials could describe a detailed walk through of what the autoML does at each step. At minimum this could also be clearly addressed in the software documentation on GitHub.

-----------------------------------------------------

--------------- Our Response -----------------

-----------------------------------------------------

Indeed, figure S11 (previously S10) does not offer details regarding the autoML pipeline, but instead focuses on the algorithms and features provided by MLme.

However, figure 2 comprehensively outlines the autoML pipeline, with the figure caption explaining all the steps involved and in what order. Furthermore, in response to your suggestion, we have expanded the information on the autoML pipeline and included it in the supplementary text. Please refer to the supplementary file (2nd page). We hope that this adequately addresses the reviewer's concerns.

###################################

######## Reviewer comment #########

###################################

3. While I understand the need for brevity, I think the addition of a sentence that indicates specifically what AutoML tools are most similar to MLme is a reasonable request that better places MLme in the context of the greater AutoML research space.

-----------------------------------------------------

--------------- Our Response -----------------

-----------------------------------------------------

We are grateful for the reviewer's agreement with keeping it concise. To address the reviewer's concerns, we have included the text in the main file, citing the names of other comparable tools. We hope that this adequately addresses the reviewer's concerns. Please refer to line 393-395.
