# Supplementary material for: Machine Learning Made Easy (MLme): a comprehensive toolkit for machine learning–driven data analysis
Source: Gigascience. 2024 Jan 11;13:giad111. doi: 10.1093/gigascience/giad111 (PMC10783149; doi:10.1093/gigascience/giad111)
Supplement: giad111_Response_to_Reviewer_Comments_Revision_3 [file giad111_response_to_reviewer_comments_revision_3.pdf]

Author's Response To Reviewer Comments

Here are the revised files
