# Supplementary material for: Machine Learning Made Easy (MLme): a comprehensive toolkit for machine learning–driven data analysis
Source: Gigascience. 2024 Jan 11;13:giad111. doi: 10.1093/gigascience/giad111 (PMC10783149; doi:10.1093/gigascience/giad111)
Supplement: giad111_Reviewer_1_Report_Original_Submission — Joe Greener -- 9/1/2023 Reviewed [file giad111_reviewer_1_report_original_submission.pdf]

Reviewer Report

Title: Machine Learning Made Easy (MLme): A Comprehensive Toolkit for Machine Learning-Driven Data Analysis

Version: Original Submission **Date:** 9/1/2023

Reviewer name: Joe Greener

Reviewer Comments to Author:

Akshay et al. present MLme, a toolkit for exploring data and automatically running machine learning (ML) models. This software could be useful for those with less experience in ML. I believe it is suitable for publication provided the following points are addressed.# Major1. The performance of models is consistently over 90% but without a reference point it is unclear how good this is. Are there results from previous studies on the same data that can be compared to, with a table comparing accuracy with MLme to previous work? Otherwise it is unclear whether MLme is supposed to be a quick way to have a first go at prediction on the data or can entirely replace manual model refinement.2. With any automated ML system it is important to impress upon users the risks of ML. For example, the splitting of data into training and test sets is done randomly, but there are cases where this is not appropriate as it will lead to data leakage between the training and test sets. This could be mentioned in the manuscript and somewhere on the GUI. There isn't really a replacement for domain knowledge, and users of MLme should have this in mind when using the software.# Minor3. More experienced ML users may want to use the software to have a first go at prediction on the data. For these users it may be useful to provide access to commands or scripts, or at least information on which functions were used, as additional options in the GUI. Users could then run these scripts themselves to tweak hyperparameters etc.4. The visualisation tab lacks an info button by the file upload to say what the file format should be.

Level of Interest

Please indicate how interesting you found the manuscript: Choose an item.

Quality of Written English

Please indicate the quality of language in the manuscript: Choose an item.

Declaration of Competing Interests

Please complete a declaration of competing interests, considering the following questions:

- Have you in the past five years received reimbursements, fees, funding, or salary from an organisation that may in any way gain or lose financially from the publication of this manuscript, either now or in the future?

- Do you hold any stocks or shares in an organisation that may in any way gain or lose financially from the publication of this manuscript, either now or in the future?
- Do you hold or are you currently applying for any patents relating to the content of the manuscript?
- Have you received reimbursements, fees, funding, or salary from an organization that holds or has applied for patents relating to the content of the manuscript?
- Do you have any other financial competing interests?
- Do you have any non-financial competing interests in relation to this paper?

If you can answer no to all of the above, write 'I declare that I have no competing interests' below. If your reply is yes to any, please give details below.

I declare that I have no competing interests.

I agree to the open peer review policy of the journal. I understand that my name will be included on my report to the authors and, if the manuscript is accepted for publication, my named report including any attachments I upload will be posted on the website along with the authors' responses. I agree for my report to be made available under an Open Access Creative Commons CC-BY license (http://creativecommons.org/licenses/by/4.0/). I understand that any comments which I do not wish to be included in my named report can be included as confidential comments to the editors, which will not be published.

Choose an item.

To further support our reviewers, we have joined with Publons, where you can gain additional credit to further highlight your hard work (see: https://publons.com/journal/530/gigascience). On publication of this paper, your review will be automatically added to Publons, you can then choose whether or not to claim your Publons credit. I understand this statement.

Yes Choose an item.
