# Supplementary material for: Machine Learning Made Easy (MLme): a comprehensive toolkit for machine learning–driven data analysis
Source: Gigascience. 2024 Jan 11;13:giad111. doi: 10.1093/gigascience/giad111 (PMC10783149; doi:10.1093/gigascience/giad111)
Supplement: giad111_Reviewer_2_Report_Original_Submission — Ryan J. Urbanowicz -- 9/5/2023 Reviewed [file giad111_reviewer_2_report_original_submission.pdf]

Reviewer Report

Title: Machine Learning Made Easy (MLme): A Comprehensive Toolkit for Machine Learning-Driven Data Analysis

Version: Original Submission **Date:** 9/5/2023

Reviewer name: Ryan J. Urbanowicz

Reviewer Comments to Author:

In this paper the authors introduce MLme, a comprehensive toolkit for machine-learning driven analysis. The authors discuss the benefits and limitations of their toolkit and provide a demonstration evaluation on 6 datasets suggesting it's potential value. Overall MLme seems like a nice, easy to use tool with a good deal of potential and value. However as the developer of STREAMLINE, an AutoML toolkit with a number of very similar goals and design architecture to MLme it was very surprising to have it not referenced or compared to in this paper. My major concerns involve the limited details about what this specifically does/includes (e.g. what 16 ML algorithms are built in), as well as what seems like a limited and largely biased comparison of this toolkit's capabilities to other autoML tools (most specifically STREAMLINE which has a significant degree of similarity).- There are many other autoML tools out there that that authors have not considered in their Table S1 or referenced. Eg. MLBox, AutoWeka, H20, Devol, Auto-Keras, TransmorgriffAI, and most glaringly in for this reviewer, STREAMLINE (https://github.com/UrbsLab/STREAMLINE).- In particular, with respect to STREAMLINE (https://link.springer.com/chapter/10.1007/978-981-19-8460-0_9), there are a large number of pipeline similarities and a similar analysis mission/goals to MLme that make it extremely relevant to cite and contrast to in this manuscript as well as in Table S1. STREAMLINE has a similar focus on the end-to-end ML analysis pipeline including automated exploratory analysis, data processing, feature selection, ML modeling with 16 algorithms, evaluation, and results visualization generation, interactive visualizations, pickled output storage, etc. The first STREAMLINE paper was published March of 2023, and a preprint of that manuscript published June 2022, as well as a precursor implementation of this pipeline published as a preprint in Aug of 2020 (https://arxiv.org/abs/2008.12829). This in contrast with MLme's preprint published July of 2023. While MLme has a number of potentially nice features that STREAMLINE does not (i.e. a GUI interface, spider plots, easy color palate selection, inclusion of a dummy classifier, ability to handle multi-class classification [which is not yet available, but in development for STREAMLINE along with regression]), it lacks other potentially important features that STREAMLINE does have (i.e. automated hyperparameter optimization, basic data cleaning and feature engineering [in the newest release], collective feature selection, pickled models for later reuse, collective feature importance visualizations, a pdf analysis summary report, the ability to quickly evaluate models on new replication data, and potentially other capabilities that I can't highlight because of limited details on what MLme includes). The absence of hyperparameter optimization is a particularly problematic omission from MLme, as this a fairly critical element of any machine learning analysis pipeline.-Table S1 should be expanded to highlight a broader range of toolkit features to better highlight the strengths and weaknesses of a greater variety of methodologies. The present table seems a bit cherry picked to make

MLme stand out as appearing to have more capabilities than other tools, but there are uncaptured advantages to these other approaches.-This manuscript includes no citations justifying their pipeline design choices. In particular, I'm most concerned with the author's justification of automatically including data resampling by default as it is well known that this can introduce bias in modeling. It's also not clear what determines if data resampling is required, and whether this only impacts training data or also testing data.- Its not clear that resampling is a good/reliable strategy for an automated machine learning framework since data resampling to create a more balanced dataset can also incorporate bias in to an ML model.- In the context of potential datasets from different domains (including biomedical data), the datasets identified in this paper as being "large" have only up to 1500 sample and only up to 5520 features, which would not be considered large by most data scientist standards.- There are largely limited details in this paper and the software's github documentation in terms of transparently indicating exactly what this pipeline does, and what options, algorithms, evaluation metrics, and visualizations it includes.- Since the authors do not benchmark MLme against any other autoML tool and they have a very limited set of benchmarked datasets (6 total, with limited diversity of data types, sizes, feature types), I don't think it's fair to claim that their methodology necessarily excels in it's core objective of addressing classification tasks. Ideally the authors would conduct benchmarking comparisons to STREAMLINE, as well as other autoML toolkits, however this might also understandably be outside the scope of this current paper. I do suggest the authors be more conservative in what assertions they make and conclusions they draw with respect to MLme. The authors might consider using established ML or AutoML benchmark benchmark datasets used by other algorithms and frameworks to compare or facilitate comparison of their pipeline toolkit to others.

Level of Interest

Please indicate how interesting you found the manuscript: Choose an item.

Quality of Written English

Please indicate the quality of language in the manuscript: Choose an item.

Declaration of Competing Interests

Please complete a declaration of competing interests, considering the following questions:

- Have you in the past five years received reimbursements, fees, funding, or salary from an organisation that may in any way gain or lose financially from the publication of this manuscript, either now or in the future?
- Do you hold any stocks or shares in an organisation that may in any way gain or lose financially from the publication of this manuscript, either now or in the future?
- Do you hold or are you currently applying for any patents relating to the content of the manuscript?

- Have you received reimbursements, fees, funding, or salary from an organization that holds or has applied for patents relating to the content of the manuscript?
- Do you have any other financial competing interests?
- Do you have any non-financial competing interests in relation to this paper?

If you can answer no to all of the above, write 'I declare that I have no competing interests' below. If your reply is yes to any, please give details below.

I have no financial competing interests. Non-financial competing interests: We have developed, previously published on, and are conducting ongoing extensions to an automated machine learning analysis pipeline with similar goals and a similar pipeline architecture to MLme, called STREAMLINE.

I agree to the open peer review policy of the journal. I understand that my name will be included on my report to the authors and, if the manuscript is accepted for publication, my named report including any attachments I upload will be posted on the website along with the authors' responses. I agree for my report to be made available under an Open Access Creative Commons CC-BY license (http://creativecommons.org/licenses/by/4.0/). I understand that any comments which I do not wish to be included in my named report can be included as confidential comments to the editors, which will not be published.

Choose an item.

To further support our reviewers, we have joined with Publons, where you can gain additional credit to further highlight your hard work (see: https://publons.com/journal/530/gigascience). On publication of this paper, your review will be automatically added to Publons, you can then choose whether or not to claim your Publons credit. I understand this statement.

Yes Choose an item.
