# Supplementary material for: Machine Learning Made Easy (MLme): a comprehensive toolkit for machine learning–driven data analysis
Source: Gigascience. 2024 Jan 11;13:giad111. doi: 10.1093/gigascience/giad111 (PMC10783149; doi:10.1093/gigascience/giad111)
Supplement: giad111_Reviewer_2_Report_Revision_1 — Ryan J. Urbanowicz -- 10/9/2023 Reviewed [file giad111_reviewer_2_report_revision_1.pdf]

Reviewer Report

Title: Machine Learning Made Easy (MLme): A Comprehensive Toolkit for Machine Learning-Driven Data Analysis

Version: Revision 1 **Date:** 10/9/2023

Reviewer name: Ryan J. Urbanowicz

Reviewer Comments to Author:

Overall I think the authors have made some good improvements to this paper, although it does not seem like the main body of the paper has changed much with most of the updates going into supplemental materials. However, I think this work is worthy of publication once the following items are addressed. (which I still feel strongly should be addressed, but should be fairly easy to do so).1. Limitations section: While the authors added some basic comparisons to a few other AutoML tools, I do not see how they are justified in saying that MLme 'excells' in it's core objective of addressing classification tasks. This implies it is better performing a classification than other methods, which is not at all backed up here, and indeed would be very difficult to prove as it would require a huge amount of analyes over a broad range of simulated and real world benchmark datasets, and incomparison to many or all orther other autoML tools. At best i think the authors can say here that it is at least comparable in performance to AutoML tools (X, Y, Z) in its ability to conduct classification analyses. And according to Figure S9 this is only across 7 datasets, and focused only on the F1 score which could also be missleading or cherry picked. At best I believe the authors can say in the paper that "Initial evaluation across 7 datasets suggested that MLMe performed comparably to TPOT and Hyperopt-sklearn with respect to F1 score performance. This suggests that MLme is effective as an automated ML tool for classification tasks. " (or something similar).2. While the authors lengthened the supplemental materials table comparing ML algorithms (mainly by adding some other autoML tools, this table is intentionally presenting the capabilities of tools in a way that make it appear like MLme does the most (with the exception of the 'features' column) . For example, what about a column to indicate if an autoML tool has an automated pipeline discovery component (like TPOT)? In terms of AutoML, this table is structured to highlight the benefits of MLme, rather than give a fair comparison of AutoML tools (which is my major concern here). In terms of AutoML performance and usability there is alot more to these different tools than the 6 columns presented. In this table 'features' seems like an afterthought, but is arguably the most important aspect of an AutoML.3. Additionally, the information presented in the autoML comparison table does not seem to be entirely accurate, or at least how the columns are defined is not made entirely clear. Looking at STREAMLINE, which can be run by users with no coding experience (as a google colab notebook), it has a code free option (just not a GUI), STREAMLINE also generates more than two exploratory analysis plots, and more results visualizations plots than indicated). While I agree that MLme has many more ease of use functionality in comparison to STREAMLINE (which is a very nice plus), a reader might look at this table and think they need to know how to code in order to use STREAMLINE, which is not the case. Could the authors at least define their criteria for the "code free" column. As it's presented now it seems to be the same exact criteria as for GUI (in which case this is

redundant). The same is true for the legend for the table where '*' indicates that coding experience is required for designing a custom pipeline. This requires more clarification, as STREAMLINE can be customized easily without coding experience by simply changing options in the Google Colab notebook, and TPOT automatically discovers new analysis pipelines which isn't reflected at all.4. While I appreciate the authors adding a citation for STREAMLINE and some other autoML tools not previously cited, it would be nice for the authors to discuss other AutoML tools further in their main paper, as well as to acknowledge in the main paper which AutoML tools are most similar to MLme in overall design and capabilities. Based on my own review of AutoML tools the most similar tools would include STREAMLINE and MLIJAR-supervised.5. I like the addition of Figure S10 that more clearly lays out the elements included in MLme, but I still think the paper and documentation lacks a clear and transparent walk through of exactly what happens to the data and how the analyses are conducted from start to finish when using the AutoML (at least by default). This is important to trusting what happens under the hood for reporting results, etc.Other comments responding to author responses:* I still disagree with the authors that a dataset with up to 1500 samples or up to 5520 features could be considered large by today's standards across all research domains. Even within biomedical data, datasets up to 100K subjects are becoming common, and 'omics' datasets regularly reach hundreds of thousands to multiple millions of features. I am glad to see the authors adding a larger dataset, but i would still be cautions when making suggestions about how well MLme handles 'large' datasets without including specifics for context. However ultimately this is subjective, and not preventing me from endorsing publication.* I also disagree that MLme isn't introducing a new methodology. The steps comprising an AutoML tool can be considered in itself a new methodology, even if it is built on established components, because there are still innumerable ways to put a machine learning analysis pipeline together that adds bias, data leakage, or just yields poorer performance. Thus I also don't think it's fair to just 'assume' your method will work as well as other AutoML tools, especially when you've ran it on a limited number of datasets/problems.

Level of Interest

Please indicate how interesting you found the manuscript: Choose an item.

Quality of Written English

Please indicate the quality of language in the manuscript: Choose an item.

Declaration of Competing Interests

Please complete a declaration of competing interests, considering the following questions:

- Have you in the past five years received reimbursements, fees, funding, or salary from an organisation that may in any way gain or lose financially from the publication of this manuscript, either now or in the future?

- Do you hold any stocks or shares in an organisation that may in any way gain or lose financially from the publication of this manuscript, either now or in the future?
- Do you hold or are you currently applying for any patents relating to the content of the manuscript?
- Have you received reimbursements, fees, funding, or salary from an organization that holds or has applied for patents relating to the content of the manuscript?
- Do you have any other financial competing interests?
- Do you have any non-financial competing interests in relation to this paper?

If you can answer no to all of the above, write 'I declare that I have no competing interests' below. If your reply is yes to any, please give details below.

I have no financial interests, but I am an AutoML methods developer (i.e. STREAMLINE).

I agree to the open peer review policy of the journal. I understand that my name will be included on my report to the authors and, if the manuscript is accepted for publication, my named report including any attachments I upload will be posted on the website along with the authors' responses. I agree for my report to be made available under an Open Access Creative Commons CC-BY license (http://creativecommons.org/licenses/by/4.0/). I understand that any comments which I do not wish to be included in my named report can be included as confidential comments to the editors, which will not be published.

Choose an item.

To further support our reviewers, we have joined with Publons, where you can gain additional credit to further highlight your hard work (see: https://publons.com/journal/530/gigascience). On publication of this paper, your review will be automatically added to Publons, you can then choose whether or not to claim your Publons credit. I understand this statement.

Yes Choose an item.
