# Supplementary material for: Machine Learning Made Easy (MLme): a comprehensive toolkit for machine learning–driven data analysis
Source: Gigascience. 2024 Jan 11;13:giad111. doi: 10.1093/gigascience/giad111 (PMC10783149; doi:10.1093/gigascience/giad111)
Supplement: giad111_Reviewer_2_Report_Revision_2 — Ryan J. Urbanowicz -- 10/27/2023 Reviewed [file giad111_reviewer_2_report_revision_2.pdf]

Reviewer Report

Title: Machine Learning Made Easy (MLme): A Comprehensive Toolkit for Machine Learning-Driven Data Analysis

Version: Revision 2 **Date:** 10/27/2023

Reviewer name: Ryan J. Urbanowicz

Reviewer Comments to Author:

At this point I earnestly wish to see this paper published, and in acknowledging my own potential bias as a developer of STREAMLINE and participant in the development of TPOT, I am still recommending minor revision. At minimum, for me to recommend acceptance of this paper the following small but critical issue needs to be addressed, otherwise I must recommend reject. I believe this concern is well justified by scientific standards. I also still strongly recommend the authors re-consider the other non-critical issues reiterated below as a way to make their paper stronger and to be better received by the scientific community. If the journal editor disagrees with my assessment, I would still be happy to see this work published, however I must stand by my assertions below.Critical Issue:Limitations section: The authors updated the text - "excells in it's core objective of addressing classification tasks." To "it excels in its primary objective of addressing pipeline development for classification tasks.The use of the word 'excells' is the key problem, as this word is defined as "to do or be better than others". While the change in phrasing correctly no longer implies that MLme performed better than the other evaluated AutoML tools, it does still imply that it is the best in developing a pipeline for classification tasks, but no specific evidence is provided in the paper to support this assertion. I.e. there were no studies comparing how easy the tool was for users to apply than other autoML, and no detailed comparison of what pipeline elements could be included by MLme vs other autoML or pipeline development tools. The fact that MLme doesn't include hyperparameter optimization is in itself a limitation that I think would prevent MLme from being claimed as excelling or superior in pipeline development to other tools/platforms, even if it's easier to use that other tools. As phrased in the reviewer response, the authors could say that MLme is well-equipped to handle pipeline development as this would be a fair statement. All together I'd strongly encourage the authors not to make statements about the superior aspects of MLme without clearly backing up these statements with direct comparisons. Instead I'd suggest highlighting elements of MLme that are 'unique' or provide more functionality in contrast with other tools. In the reviewer response the authors make the claim that MLme is superior in terms of ease of use for visualization and exploratory analysis. If they want to make that statement in the paper backed up by accurate comparisons to other tools, I'd agree with that addition.Non-Critical Issues that I feel still should be addressed:1. Table S1 has been updated to remove the inaccuracies I previously pointed out, however this alone does not change the broader concern I had regarding the intention of this table (which is to highlight the parts of MLme that appear better than other AutoML tools without fairly pointing out the limitations of MLme in contrast with other tools). As a supplemental materials table, I do not feel this is critical, but I think making a table that more fairly reflects strengths and limitations of different tools would greatly strengthen this paper.2. The pipeline design in Figure 2 and and S10 are both high-level

and still do not provide enough detail/clarity to understand exactly what happens and in what order when applying the autoML element of MLme. They key words here being transparency and reproducibility. The supplemental materials could describe a detailed walk through of what the autoML does at each step. At minimum this could also be clearly addressed in the software documentation on GitHub.3. While I understand the need for brevity, I think the addition of a sentence that indicates specifically what AutoML tools are most similar to MLme is a reasonable request that better places MLme in the context of the greater AutoML research space.

Level of Interest

Please indicate how interesting you found the manuscript: Choose an item.

Quality of Written English

Please indicate the quality of language in the manuscript: Choose an item.

Declaration of Competing Interests

Please complete a declaration of competing interests, considering the following questions:

- Have you in the past five years received reimbursements, fees, funding, or salary from an organisation that may in any way gain or lose financially from the publication of this manuscript, either now or in the future?
- Do you hold any stocks or shares in an organisation that may in any way gain or lose financially from the publication of this manuscript, either now or in the future?
- Do you hold or are you currently applying for any patents relating to the content of the manuscript?
- Have you received reimbursements, fees, funding, or salary from an organization that holds or has applied for patents relating to the content of the manuscript?
- Do you have any other financial competing interests?
- Do you have any non-financial competing interests in relation to this paper?

If you can answer no to all of the above, write 'I declare that I have no competing interests' below. If your reply is yes to any, please give details below.

No financial competing interests. However I am am similarly a developer of AutoML tools.

I agree to the open peer review policy of the journal. I understand that my name will be included on my report to the authors and, if the manuscript is accepted for publication, my named report including any attachments I upload will be posted on the website along with the authors' responses. I agree for my report to be made available under an Open Access Creative Commons CC-BY license (http://creativecommons.org/licenses/by/4.0/). I understand that any comments which I do not wish to

be included in my named report can be included as confidential comments to the editors, which will not be published.

Choose an item.

To further support our reviewers, we have joined with Publons, where you can gain additional credit to further highlight your hard work (see: https://publons.com/journal/530/gigascience). On publication of this paper, your review will be automatically added to Publons, you can then choose whether or not to claim your Publons credit. I understand this statement.

Yes Choose an item.
